# An Overview of Biopolymeric Electrospun Nanofibers Based on Polysaccharides for Wound Healing Management

**DOI:** 10.3390/pharmaceutics12100983

**Published:** 2020-10-17

**Authors:** Andreea-Teodora Iacob, Maria Drăgan, Oana-Maria Ionescu, Lenuța Profire, Anton Ficai, Ecaterina Andronescu, Luminița Georgeta Confederat, Dan Lupașcu

**Affiliations:** 1 Department of Pharmaceutical Sciences, Faculty of Pharmacy, University of Medicine and Pharmacy “Grigore T. Popa” Iași, 700115 Iasi, Romania; andreea.panzariu@umfiasi.ro (A.-T.I.); maria.wolszleger@umfiasi.ro (M.D.); oana-maria.dc.ionescu@d.umfiasi.ro (O.-M.I.); dan.lupascu@umfiasi.ro (D.L.); 2 Department of Science and Engineering of Oxide Materials and Nanomaterials, Faculty of Applied Chemistry and Materials Science, University Politehnica of Bucharest, 060042 Bucuresti, Romania; anton.ficai@upb.ro; 3 Academy of Romanian Scientists, Ilfov st 3, 050085 Bucharest, Romania; 4 Department of Preventive Medicine and Interdisciplinarity, Faculty of Medicine, University of Medicine and Pharmacy “Grigore T. Popa” Iași, 700115 Iasi, Romania; georgeta-luminita.confederat@umfiasi.ro

**Keywords:** polysaccharides, chitosan, hyaluronic acid, alginates, gums, pectins, cellulose, starch, dextran, pullulan, electrospun nanofibers, wound healing, wound dressings, electrospinning

## Abstract

Currently, despite the thoroughgoing scientific research carried out in the area of wound healing management, the treatment of skin injuries, regardless of etiology remains a big provocation for health care professionals. An optimal wound dressing should be nontoxic, non-adherent, non-allergenic, should also maintain a humid medium at the wound interfacing, and be easily removed without trauma. For the development of functional and bioactive dressings, they must meet different conditions such as: The ability to remove excess exudates, to allow gaseous interchange, to behave as a barrier to microbes and to external physical or chemical aggressions, and at the same time to have the capacity of promoting the process of healing by stimulating other intricate processes such as differentiation, cell adhesion, and proliferation. Over the past several years, various types of wound dressings including hydrogels, hydrocolloids, films, foams, sponges, and micro/nanofibers have been formulated, and among them, the electrospun nanofibrous mats received an increased interest from researchers due to the numerous advantages and their intrinsic properties. The drug-embedded nanofibers are the potential candidates for wound dressing application by virtue of: Superior surface area-to volume ratio, enormous porosity (can allow oxy-permeability) or reticular nano-porosity (can inhibit the microorganisms’adhesion), structural similitude to the skin extracellular matrix, and progressive electrospinning methodology, which promotes a prolonged drug release. The reason that we chose to review the formulation of electrospun nanofibers based on polysaccharides as dressings useful in wound healing was based on the ever-growing research in this field, research that highlighted many advantages of the nanofibrillary network, but also a marked versatility in terms of numerous active substances that can be incorporated for rapid and infection-free tissue regeneration. In this review, we have extensively discussed the recent advancements performed on electrospun nanofibers (eNFs) formulation methodology as wound dressings, and we focused as well on the entrapment of different active biomolecules that have been incorporated on polysaccharides-based nanofibers, highlighting those bioagents capable of improving the healing process. In addition, in vivo tests performed to support their increased efficacy were also listed, and the advantages of the polysaccharide nanofiber-based wound dressings compared to the traditional ones were emphasized.

## 1. Introduction

The skin is the outermost stratum of the human body that serves as a barrier protecting the body’s internal medium from the external one. Additionally, skin plays key roles in various body functions such as in sensing and detection, in adjusting body temperature with water waste control, and in supporting blood vessels and nerves, where any skin damage will result in malfunctioning of the activities mentioned above [1]. It is well known that all living organisms regenerate as part of natural processes to preserve tissues and/or organs, but mammals have restricted regenerative abilities, including forming thick scars in skin and tissues, which supports the healing process [2]. Therefore, the complex process of damaged tissues restoration involves several overlapping steps: Influx of inflammatory cells and collagen formation, cytokine actions, depositions of the extracellular matrix (ECM) and cellular reorganization with scar appearance [3,4]. Despite numerous researches being conducted in recent years aiming at the formulation of wound dressing biomaterials, no currently available material fulfills the needed characteristics for a speedy and improved recovery of injured tissues. Therefore, in the domain of tissue-engineering, the search for an ideal wound-dressing skin substitute based on different biomaterials remains a challenge, and nanotechnology offers an alternative worth considering, especially when using biomaterials from the polysaccharide class [5].

The rising knowledge in wound pathophysiology and etiology resulted in an influx of ground-breaking medical technologies/nanotechnologies into the conventional wound healing field and consequently, the global market is predicted to reach $22 bilion by 2022 [6]. Among the dressing formulation techniques, electrospinning occupies a leading place, due to its simplicity but also to its flexibility, advantages that allow the use of a wide range of biomaterials. Formhals first claimed in 1934 the patent of high-voltage electrostatic spinning process, a moment that can be considered as the origin of electrospinning. In the last 20 years, relevant and convincing studies on electrospun micro/nano fibers and electrospinning process have been performed, in which the electrospinning process was extensively described ranging from the mechanism explanation to the applications of electrospun fibers [7,8]. The following predominant advantages can be deduced from the comparison made with the conventional methods used for the nanofibers’ formulation: Simple preparation with a wide choice of materials, low cost, and good flexibility, advantages that entitle the further studies of these types of materials [9]. Also, the electrospun nanofibers (eNFs) possess specific properties, including large surface areas, high porosities, changeable morphologies, and controllable mechanical properties. These features can be customized according to the distinctive drug delivery necessities of various applications [9,10].

The aim of this review is to emphasize the important role of research in the nanotechnology field of wound healing and to identify novel drug systems technologies that can both improve the regenerative capacity of human tissues and that can combat the occurrence of complications such as fibrosis and sepsis. The present review’s primary objective consists of the collection and critical analysis of data derived from recent and ongoing research conducted in the field of advanced drug delivery in wound healing using eNFs as dressing materials based on polysaccharides.

## 2. Wound Healing

The appearance of wounds can occur only after the skin barrier is destroyed by physical, chemical, microbial, or immunological agents [1]. Skin lesions are inexorable events in patients’ life and because the most common complication that can occur is represented by the endogenous bacterial infection, wound treatment is imperative [11]. A dressing considered ideal should meet a number of characteristics such as the following: Acting as a barrier towards microorganisms’ contamination, preserving a moist medium at the wound site, permitting gaseous exchange, and also removing the surplus of exudate. Other desirable features for a wound dressing refer to the ability to be non-allergenic, non-toxic, non-adherent, and effortlessly pulled out without damage, and also it should be formulated from a readily available bio-compound, which involves limited processing, has antibacterial properties, and can also enhance wound healing [12].

### 2.1. Phases of Wound Healing

**Figure 1:**
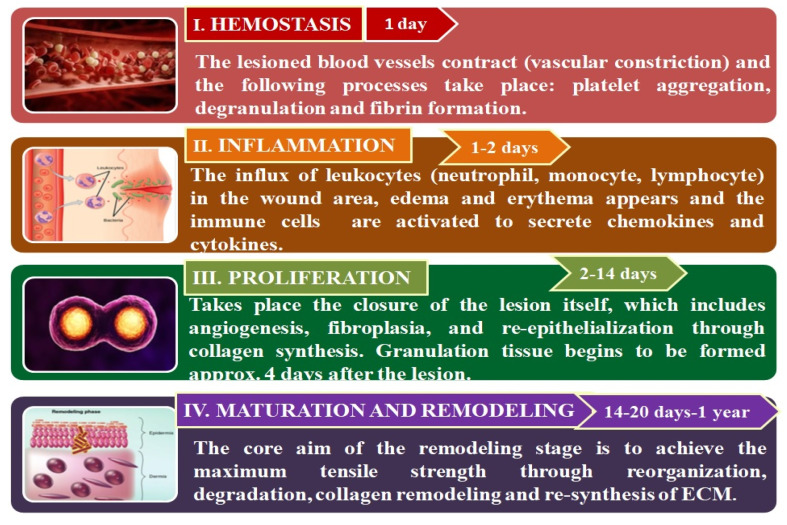
The representation of 4 stages of wound healing [15].

Wound healing is a complicated, multifaceted process governed by sequential, but in the same time, overlapping stages: Hemostasis, inflammation, proliferation, and remodeling [13] as shown in Figure 1. These phases and their physiological roles must take place at a specific time, in a proper sequence, and must persist for an optimal duration at an adequate intensity [14].

#### 2.1.1. Hemostasis

The etymology of the word hemostasis comes from the juxtaposition of two Latin words derived from the ancient Greek, “haimo”, which means blood and “stasis”, which represents the action of stopping. Hemostasis is an intricate and immediate reaction towards the damaged blood vessel from a wound in order to stop the blood loss via vasoconstriction and plug formation [16]. After a skin injury takes place, the exposed sub-endothelium and tissue factor will stimulate platelet accumulation, which will result in degranulation and releasing of chemotactic factors (chemokines) and growth factors (GFs) necessary for the clot formation, with this succession of steps constituting the hemostasis process [17]. The thrombocyte is able to secrete different proteins such as sphingosine-1-phosphate, von Willebrand factor (vWF), fibronectin, and thrombospondin, in order to enhance the activation of growth factors and thrombocytes such as transforming growth factors (TGF-α, TGF-β), platelet-derived growth factor (PDGF), insulin-like growth factor (IGF), and interleukin 1 (IL-1), in order to sustain in the post-hemostasis phases of the wound healing (Bielefeld et al., 2013). Within the complex healing process, other growth factors such as EGF (epidermal growth factor), VEGF (vascular endothelial growth factor), and FGF (fibroblast growth factor) intervene. Consequently, EGF overall promotes wound healing first by activating the epidermal growth factor receptor (EGFR) signaling path [18], which subsequently will lead to an enhanced cell migration (keratinocyte, endothelial cells, and fibroblast) at the injured area, and also will promote the angiogenesis and proliferation [19]. The main role played by VEGF is in the process of angio- and/or vasculo-genesis, essential in wound healing, but also it contributes significantly to the improvement of collagen deposition and re-epithelialization. Hence, this subtype of growth factor will improve the vascular permeability and the angiogenesis by stimulating endothelial cell migration and dissemination and by permitting the penetration of inflammatory cells into the wound area [20]. FGFs are a large group of secretory cytokines known to possess a powerful chemo-attractant and mitogenic action towards the endothelial cells, participating in their recruitment, differentiation, and dissemination. Accordingly, FGF will regulate the influx and differentiation of cells with mesodermal, endodermal, or ectodermal origin, will promote cell proliferation, and also will play a key role in post injury recovery [21].

The fibrin network performs multiple roles such as: Forming a barrier against the microorganisms’ invasion, reestablishing homeostasis, organizing the necessary momentary matrix for cell migration, which in turn restores the skin’s integrity and reassures the function of protective barrier against the external environment [15].

#### 2.1.2. Inflammation

The wound swelling caused by the body fluid’s accumulation is one of the first signs of inflammation. Neutrophils are the first cells to emerge at the wound area, cells that clean out debris and bacteria in order to supply a proper environment for wound healing process. The next step consists of the macrophage accumulation, which will facilitate phagocytosis of bacteria and of the damaged tissue [22]. During the inflammatory process, cytokine-secreting macrophage, and natural killer (NK) cells are attracted to the injury location and orchestrates the elimination of the invading pathogens, while for stopping further bleeding, the secreted PDGF will simultaneously coordinate the thrombin complex activation [23]. In the phase of inflammation, cytokines (such as IL-1, IL-6) and macrophages (like PDGF, TNF-α, tumor necrosis factor-α, TGF-β) produce growth factors that will ease later the post-inflammation stage of proliferation for the endothelial cells and fibroblasts [16].

#### 2.1.3. Proliferation

Fibroplasia and the revascularization of the wound take place at the same time, and the combination of migration and proliferation results in the formation of angiogenesis process. Angiogenesis emerges at the site of vessels near to the wound and consists of stimulating the migration of endothelial cells [4]. The mediators for chemotaxis and endothelial cell growth are the cytokines produces by macrophages, platelets, and lymphocytes from the wound location [24]. The endothelial cells proliferation takes place when the proteolytic enzymes produced by the activated endothelial cells will dissolve the basal lamina, a stage known as sprouting. Smooth muscle cells and pericytes help stabilize vessels walls and with the onset of blood flow the phase of angiogenesis gets finalized [25]. 

#### 2.1.4. Maturation and Remodeling

The wound matrix constitution is represented by fibrin and fibronectin in the early wound stages, due to hemostasis and macrophages activation and accumulation. Endothelial cells, macrophages, and myofibroblasts can exit the wound area, while the remaining ones undergo the apoptosis process. The formation of other matrix components that comprise glycosaminoglycan and fibroblasts can also take place in this step, though fibrillar collagen type I (80–90%) and reticular collagen type III (10–20%) are the principal components of the intact dermis [4]. All the stages initiated in the previous steps of wound healing will finish when remodeling occurs and when the scar formation takes place [26]. The scar formation implies a type of mechanics different from normal connective tissue mechanics, where the scar tissue is liable for close liaison between dermis and epidermis, and also is immature and more pliable [27].

### 2.2. Wound Classification

**Figure 2:**
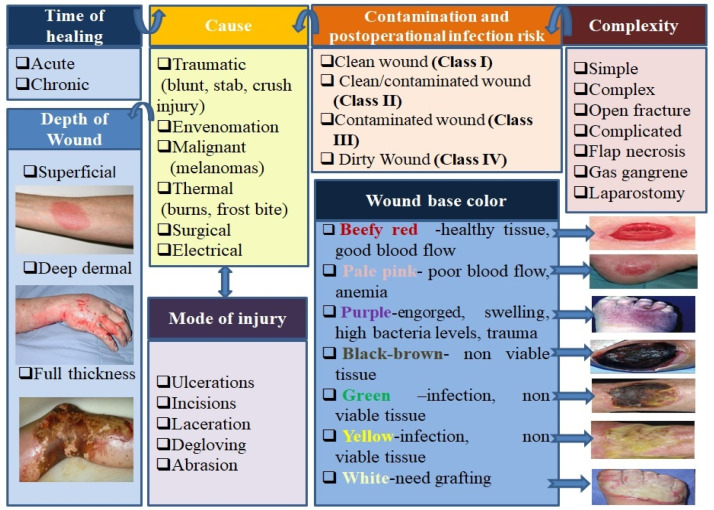
Wounds classification [28].

A skin injury produced as a result of surgical injury or an accident is considered an acute wound. Initially, all wounds can be described as acute and are anticipated to evolve through a normal process of wound healing. If one of the four steps of wound healing described above is prolonged more than six weeks, the wound can be considered as chronic (leg ulcers, pressure ulcers, etc.) [28]. Scientists have stated that the healing process of acute wounds occurs following the normal four phases described above and takes place over a period of time that can vary from 8 to 12 weeks according to the degree of impairment done to the epidermis, to the wound size, and depth. Meanwhile, the chronic wounds do not progress through the same sequence of healing stages and there is no indication at wound area of healing in a timely/orderly manner [29,30]. The acute wounds can be further classified in other categories such as mechanical (surgical or traumatic wounds), chemical or thermal injuries (burns), malignant (melanomas), etc. (Figure 2). The microorganisms’ degree of replication can determine the classification of wounds into classes of wounds as having colonization, contamination, critical colonization/local infection, or disseminated invasive infection [14].

### 2.3. Local Factors Which Influence Wound Healing

#### 2.3.1. Contamination

Once the skin is injured, the saprophytic flora, located on the surface of the skin, obtains accession to the underlying tissues. The contamination and infection remain serious complications of wounds and the sepsis is a major cause of morbidity or even mortality in patients with the critical type of wound. Wound infection occurs in a traumatized tissue medium when there is an imbalance between bacterial colonization and the host, in the favor of bacteria. Additionally, the infection at the injury site has the ability to provoke a systemic response, like sepsis, but it also enables the inhibition of the multiple phases implicated in the structured progression and evolution of normal wound healing [31]. Inflammation is a physiological stage of the wound healing process and has a crucial role in the elimination of contaminating micro-organisms, therefore when microbial clearance is incomplete due to the lack of efficient decontamination, inflammation may be prolonged. So, due to the fact that wounds are prone to bacterial/fungal contamination, inducing damages to the affected tissues, the healing process will be impaired and disrupted [32].

#### 2.3.2. Moisture 

In addition to the role of barrier against contamination with microorganisms, a proper wound dressing should maintain a moist/humid medium at the wound site, allowing a good gaseous exchange and also permitting to absorb exudates [33]. It was shown that a humid environment in wound area can accelerate wound healing by promoting re-epithelialization and reducing the inflammatory reaction. The mechanisms of wound healing that take place in a moist environment are extended presence of growth factors and proteinases, faster and easier migration of epidermal cells, as well as superior fibroblast growth and keratinocyte proliferation [29,34].

#### 2.3.3. Oxygenation 

Oxygen is vital for approximately all wound-healing processes because it plays significant roles for cell metabolism, notably in the energy production through the use of ATP (Adenosine triphosphate). It promotes wound contraction, induces angiogenesis, prevents wounds infection, enhances keratinocyte migration, differentiation, and re-epithelialization, and also it increases collagen synthesis and fibroblast proliferation.

Oxygen in the wound healing process is actively involved in the inflammatory, proliferative phase but also in the last stage of remodeling and maturation. During the inflammatory step, oxygen stimulates the bactericidal protection against pathogens, and after the pathogens are phagocytosed, oxygen is utilized via nicotinamide adenine dinucleotide phosphate (NADPH)-linked oxygenases found in leukocytes for the formation of ROS (reactive oxygen species), such as superoxide anions. Further, the superoxide anion will be subjected to reactions by which it will be transformed into other ROS such as hydroxyl radicals or hydrogen peroxide, or it will be futher used by myeloperoxidase for the formation of hypochlorous acid. All these agents formed within the phagosome have an oxidant character and are responsible of facilitating bacterial killing in wounds [35]. This formation of oxidizing structures it is also known as the respiratory burst, due to its high consumption of oxygen (approximately 98% of oxygen neutrophils-consumed is used for the respiratory burst). Thus, resistance to infection is fundamentally compromised by wound hypoxia. Additionally, the ROS act as regulator factors in the healing process, having an active role in the key stages as: Cytokine release, coagulation, cell proliferation, matrix deposition, angiogenesis, and re-epithelialization [36]. During the proliferative phase, oxygen plays the role of energy supplier for cells, owing to its participation in ATP generation, in the process of oxidative phosphorylation located in mitochondria. Furthermore, ATP will be able to induce vasodilation via plasma adenosine membrane receptors activation at the vascular endothelium and also to stimulate the immune system [37]. Finally, oxygen is required for mature collagen formation and fibroblasts accumulation during the remodeling step of wound healing. Specifically, oxygen is necessary in the hydroxylation reaction of proline and lysine from pro-collagen chains in order to stabilize the triple helices of collagen. Last but not least, another process that is oxygen dependent is represented by the wound contraction attributed to the differentiation of fibroblasts into contractile myofibroblasts, activated by TGF-β1, TGF-β2, and PDGF [38].

It has also been indicated that the healing process is severely affected in wounds where oxygenation is not re-established [14]. Depending on the time in which the wound is subjected to a decrease in oxygen concentration, the effect may be different, so temporary hypoxia may trigger the healing process, while prolonged or chronic hypoxia will lead to a delayed healing [39]. The reason that in the early wounds there is a depletion of oxygen with the onset of hypoxia is represented by vascular disturbance, but also by the fact that the metabolism of the active cells is directly involved in high oxygen consumption. Suitable oxygenation at the wound site can cause accurate healing responses and can influence, in a positive manner, the results of other treatment methods [29].

Throughout wound healing responding processes, hypoxia plays a pivotal role. In the condition of hypoxia, the release of inducible hypoxia factors (HIF) will be achieved, which induces the expression of HIF target genes with a role in counteracting the state of hypoxia. Under normal conditions of oxygenation (normoxia) the HIF-prolyl-4-hydroxylases (PHDs) hydroxylates HIF-α in an oxygen-dependent manner, thus marking it for the degradation that occurs at the level of proteosomes [40]. The PHDs under the action of various pharmacologically active substances can suffer an inhibition caused by so-called PHD inhibitors. The PHD inhibitors are favorable regulators of HIF-1, which are currently in clinical trials for treatment of some human conditions-ischemia-based [41]. Hypoxia-inducible factor-1 (HIF-1) represents the principal regulator of oxygen homeostasis and plays a pivotal role in establishing the success and the outcomes of the healing process. HIF-1 participates in all of the wound healing phases such as cell survival under hypoxic circumstances, cell migration and division, growth factor discharge, and ECM formation. We can encounter two situations, the case when HIF-1 is in deficiency or when it is in excess, both situations providing key therapeutic strategies to be used in the correlation between HIF-1 expression and pathogenesis. In the first case, when HIF-1 is deficient, then, by default, when exposed to hypoxic stimuli it would not have the capacity to respond to those stimuli, which ultimately will lead to chronic hypoxia. By contrast, in the second case of HIF-1 over-expression, an increase in the myofibroblast differentiation capacity was observed conducive to extreme matrix formation and deposition [42].

Numerous studies indicated that at early stages of wound healing, acute hypoxia promotes, via up-regulation of TGF-β, the proliferative ability of fibroblasts and consequently can sustain the initiation of wound healing, while chronic hypoxia critically reduces fibroblast action [43]. For these reasons, the chronic dermal wounds often have signs of stringent tissue hypoxia including up-regulation of the hypoxia-inducible factor pathway, which attempts to re-establish normoxia within the skin. Based on these considerations, hyperbaric oxygen treatment (HBOT) demonstrated a beneficial role in the treatment of patients with delayed wound healing [40].

## 3. Electrospun Nanofibers in Wound Healing

**Figure 3:**
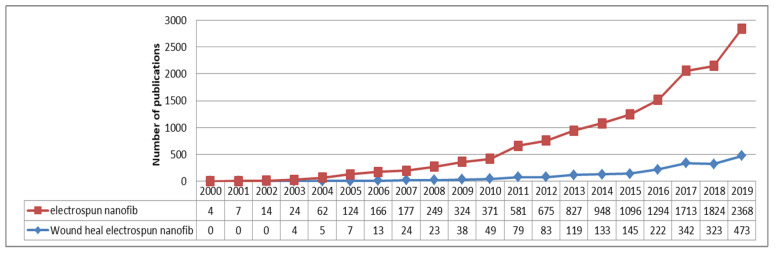
Representation of numbers of publications in the last 19 years using key-words “electrospun nanofibers” vs. “wound healing electrospun nanofibers”.

Looking on the Espacenet database of patents and searching for the key-words “electrospun nanofibers” gave approximately 350 results found in Worldwide Database and only 1 match while using the key words: “electrospun nanofibers” and “wound healing”. While searching on the Science Direct platform using key-words “electrospun nanofibers” we found that it gave a total of 12,996 articles varying by the year of publications from 1998 (1) to 2019 (2.368) with a continuous increase year after year, such as the number of publications given after the search of key-words “wound healing electrospun nanofibers” as shown in Figure 3.

### 3.1. Electrospinning

**Figure 4:**
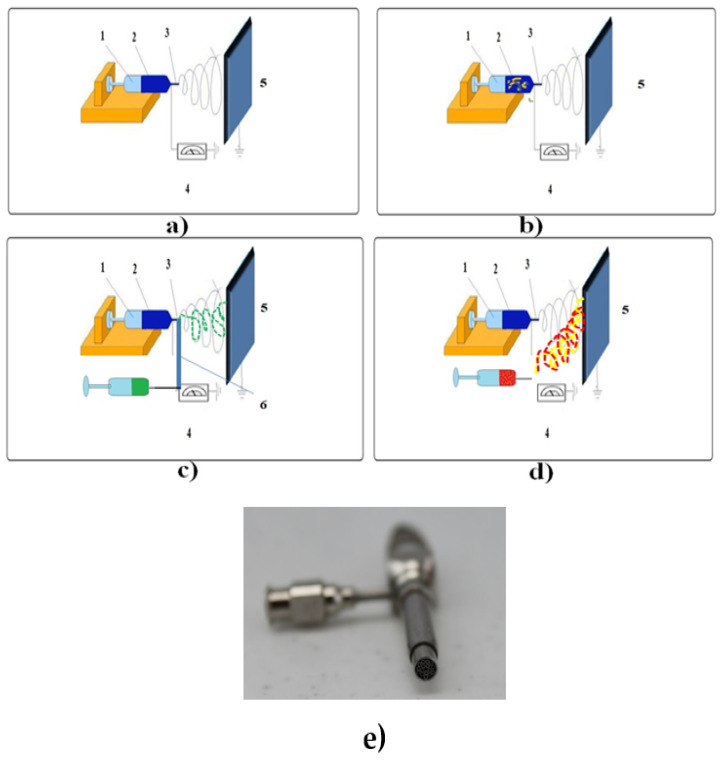
Schematic representation of different types of electrospinning process: (**a**) Blend electrospinning, (**b**) emulsion electrospinning, (**c**) co-axial electrospinning, (**d**) electrospinning-electrospraying hybrid, (**e**) spinnerrets.

Over the past several years, in the context of the ultra-rapid development of nanotechnology, the embodiment of bioactive compounds into polymer scaffolds for sustained drug release has grown into an enticing area of research [9]. Electrospinning is a cost-effective and efficient technique for fabricating steady nanofibers with diameters on the order of nanometers, ranging from 5–100 nm, which is 100–10,000 times reduced compared to the fibers produced via solution or melt spinning [33]. The uni-axial electrospinning setup includes: **1** A syringe (glass/plastic), **2** a moderate/viscid polymer solution (to be electropsun), **3** a flow rate controller, **4** a voltage producing unit, and **5** a grounded collector plate/rotating drum. The co-axial electrospinning involves the single spinneret replacement by two coaxial capillaries 6 as shown in Figure 4. During this process, through a nozzle with two concentric, capillaries can simultaneously feed two solutions, which are independently monitored by autonomous syringe pumps. Thus, in this process, two separate solutions with distinct flow-rates can be subjected to electrospinning [29]. Even though not very popular, there are multiple spinnerrets with three co-axial needles as well as spinnerrets with four or more needles disposing inside a much larger needle such as that presented in Figure 4e.

When an electric field is applied between the capillary bottom of the needle and the collecting plate, the surface load is induced by the polymeric fluid that deforms the spherical drop to a conical shape—with the appearance of the so-called “Taylor cone” [44,45]. The generation of the Taylor cone is favored by the accumulation of charge at the tip of the syringe needle, which will cause repulsion in solutions with similar charges [46]. Furthermore, the repulsive energies would surmount the surface tension of the spherical droplet and it will initiate the thread formation following the electric field direction towards the collector. By applying a high field, a charged strand of the biopolymer or synthetic polymer solution at a pre-set value can be obtained, and through solvent evaporation, nanofibers can be formulated [9,47].

Unlike other nano-materials (nanotubes, nanowires, nanorods) fabricated predominantly by bottom-up methods, electrospun nanofibers are formulated through a top-down process, where the attained steady nanofibers are relatively easy to assemble, align, and can be used in various scientific areas such as in the pharmaceutical, cosmetics, and biomedical domains of interest [48].

Regardless of the electrospinning method used in order to formulate nanofibers, three main categories of parameters should be taken into consideration:

(a) Electrospinning-related parameters (flow rate, applied voltage, needle diameter, and distance between the needle and collector);
(b) Solution-related parameters (solvent, polymer concentration, polymeric solution conductivity, and viscosity);
(c) Environmental-related parameters (humidity, temperature) [49].

In the following statements, we will treat each category of parameters separately to emphasize their role in directing the formation of electrospun fibers in the nano domain.

(a) *Electrospinning-related parameters* that influence the nano-scale orientation of the obtained fibers

The flow rate represents one of the main key-factors that influence the fiber size and size distribution. The fibrous scaffold’s diameter ranging from mm-nm is directly proportional to the solution supply rate when subjected to an electrical field. The applied voltage from the electrical field also has an enormous impact on the fibrous diameter, following a relationship of inverse proportionality between the obtained diameter and the voltage value applied at a medium solution flow rate. As increasing or decreasing the flow rate affects the nanofibers’ diameter and formation it was indicated that a minimum flow rate is preferred to preserve the balance between the release of the polymeric solution and the substitution with the next one during jet formation. Many studies have investigated the influence of the distance between the needle tip and collector and determined that large-diameter fibers are formed when the distance is small, whereas the diameter of the nanofiber shrink as the distance was augmented [50].

(b) *Solution-related parameters* that influence the nanofibers development

Regarding the polymeric solution characteristics of the solution, the conductivity and viscosity can influence the nanofibrous diameter and the diameter distribution in a considerable manner. Therefore, the high-conductivity polymeric solution will form nanofibers characterized by a wide size distribution, and at the same time the extremely low conductivity solutions combined with moderately high electrical will lead to the formation of inhomogeneous nanofibers [51]. Also related to the characteristics of the polymer solution for electrospinning, the concentration of the polymer and implicitly the viscosity of the obtained solution represent key factors in the stretching of the charged jet. To exemplify, if the concentration of the polymeric solution is low, the surface tension and applied electric field can produce the tangled polymer chains to disintegrate into fragments before reaching the collector. The elongation of the polymer jet and the comportment of the whipping jet portion have an important impact on the diameter of the nanofibers. The stretching in the whipping region due to the surface charges draws the fluid jet into the nanoscale [52].

Regarding the solvent role in the nanofibers production, different studies revealed that an ideal solvent for electrospinning process must meet the following conditions: To completely dissolve the polymers used and to have a moderate boiling point, a property that determines the volatility degree. Correspondingly, volatile solvents with moderate to high evaporation rates promote facile evaporation of the solvent from the nanofibers during their needle tip-to collector trip. At the same time, the solvents with a high degree of volatility are not being used in electrospinning process due to their high evaporation rates and low boiling points that cause the drying of the fluid jet at the needle tip, which can cause the needle tip to block [49].

(c) *Environmental parameters* tha interfere with the nanofibers’ formation

Humidity causes modifications in the nanofibers’ diameter by regulating the solidification action of the charged jet. Temperature, by affecting on the one hand the evaporation rate of the solvent and on the other hand by changing the polymeric solution’s viscosity, has the ability to orient the diameter of the fibers obtained in the nano scale.

In the co-axial electrospinning approach, the diameter of core and shell fibers can be managed by controlling and varying the specific parameters including the applied voltage and flow rate [53]. During emulsion-electrospinning, the continual phase is promptly evaporated and consequently, the viscosity is enhanced, therefore the water-base droplets comprising bioactive agents shift to the jet’s core [29].

For the electrospinning-electrospraying hybrid approach the process of electrospraying takes place concomitantly with the eNFs formation, with the advantage of eliminating the action of eNFs post-treating. In this way, it is possible to encapsulate high concentration of bioactive molecules into the eNFs’s surface by electrospraying functional bioagents onto the same collector from a distinct syringe pump [54].

#### 3.1.1. Blend Electrospinning

The most prevailing approach of blend nanofibers preparation is a facile single-step method acknowledged as blend electrospinning [55,56]. The easiest way to form these types of nanofibers is to choose a solvent in which to be soluble with both the polymer used and the bioactive substance to be incorporated. If the active substance is insoluble in the solvent used to dissolve the polymer, then the bioactive agents will be dissolved in small quantities of a different solvent followed by the polymer solution addition [57,58]. A critical aspect to take into consideration is represented by the appropriate hydrophilicity/hydrophobicity of the polymer in relation to the active bio-agents to be incorporated. The lack of solubility of bioactive compounds into the polymer solution will cause their diffusion inside the polymer and, when subjected to electrospinning, the bioactive substances will be deposited on the fiber surface resulting in undesirable burst release [59,60].

#### 3.1.2. Emulsion Electrospinning

Emulsion electrospinning is an innovative and simple approach for producing core-shell nanofibers that can be formulated in order to encapsulate functional materials (proteins, peptides, flavonoids, enzymes) [61]. In comparison with coaxial electrospinning, described below, emulsion electrospinning is a method that can develop using a single-nozzle, steady and continuous core-shell fibers. Contrary to the standard technique of blend electrospinning, in emulsion electrospinning, the drug is commonly dissolved in an aqueous solution (water phase) that is then diffused in the organic polymeric solution (oil phase) containing a suitable surfactant as emulsifier. The obtained water/oil (W/O) emulsion after subjecting it to electrospinning will form fibers or nanofibers with a core-shell construction, where the drug is embedded in the core [62]. A substantial aspect for emulsion electrospinning is represented by the stabilization of the emulsion formed, where the morphology and the features of the nanofibers are influenced by the utilized species and by the surfactants’ concentration [63].

During electrospinning process, the viscousness of the covering layer comparative to that of the inner stratum increase due to the rapidity with which the solvent evaporates from the region closer to the surface than the middle section of the polymer jet [47]. Under the pressure of a high-tension electric field, the internally motion of emulsion droplets is realized from the exterior to the center, thus the drops are compressed and strained into elliptical conformation in the axial orientation of the eNFs. A pivotal role in the misshapenness of the emulsion droplets may play the high-velocity jet subjected to braking energies generating from its interactions with the ambient air. As well, other forces including surface tension, gravity, and rheological forces can also influence the charged jet flow. The entrapment of bioactive substances has to permeate the core-shell nanofiber scaffold before reaching the exterior environment, so this method of emulsion-electrospinning can successfully escape drugs burst release without jeopardizing their bioactivity [61].

#### 3.1.3. Coaxial Electrospinning

Coaxial electrospinning represents a procedure modified from standard electrospinning, which allows the encapsulation of bioactive agents into the polymer nanofibers developing core-schell matrices [64]. In these distinct drug-delivery systems, the biomolecules embedded in the core layer are guarded against organic mediums, which can produce the bioactivity’s depletion and therapeutic efficiency’s decreasing [65]. Adjusting the shell’s thickness of the nanofibers, the drug release rate can be controlled. Henceforth, coaxial electrospinning is reputed as one of the most outstanding findings in the domain of sustained drug release [66]. The foremost advantage of the coaxial electrospinning is represented by the direct development of core-sheath designed nanofibers with concentrically aligned spinnerettes binding to distinct channels for diverse solutions [67]. This technique implies the use of two solutions, one as core solution and another one as shell solution. Next, these solutions will be put into two plastic syringes equipped with a spinneret, which can be constituted by two coaxial stainless-steel capillary needles of various diameters. The flow rate of the outer and inner fluids will be adjusted by two different syringe pumps [68]. The determining factor regarding the mechanical properties of the structures formulated following the coaxial electrospinning technique is depicted by the interaction between core and sheath, and not by the individual properties of the core polymer solution [69].

#### 3.1.4. Electrospinning-Electrospraying Hybrid

Electrospinning and electrospraying are both electro-hydrodynamic techniques, whereby applying high electric voltage, a polymeric dispersion can be spun or sprayed in order to produce fibers or particles, respectively. The standard configuration for electrospinning or electrospraying implies four important elements: A blunt-ended, stainless-steel capillary or needle, a high-voltage source power, a syringe pump, and a rotating drum or flat plate as a collector [48,70].

The electrospinning and electrospraying in a concomitant procedure have been described for the formulation of PANI/carbon nanofiber/particle network electrodes for hybrid capacitors or for the production of reactive membranes for water filtration and electrodes for fuel cells. This innovative technique distinguishes from electrospraying or electrospinning taken separately, where particles and nanofibers can shape interlinked morphologies. In conclusion, this hybrid method supplies a simple way to merge fibers/nanofibers or particles in a mixed scaffold with adjustable material loadings, fiber diameter, and particle size [71].

#### 3.1.5. Advantages of Electrospinning in Wound Healing Management

Wound dressings formulated from electrospun nanofibers exhibit favorable characteristics for the improvement of healing process. Their 3D structure imitates the skin’s architecture of extracellular matrix (ECM), which has a pivotal role in sustaining the processes of cell adhesion and proliferation [72]. The porous texture of these nanofibrillary matrices is congruent with the nutrients and gaseous exchanges, with the adsorption of the injury’s exudates, as well with the prevention of bacterial contamination, so the membrane’s architecture will contribute to adhesion, cell penetration, and proliferation [73,74].

The association of the large surface area to volume ratio of the eNFs along with the option of choosing the most suited solvent for an increase in the solubilization of bioactive compounds grants these dressing withs superior loading abilities. Furthermore, the drug loading in these nanofibrous scaffolds can be realized using distinct techniques that vary from the nature of bio-agents merging with the polymer to the encapsulation of secondary drug carriers [65,75].

### 3.2. Polysaccharides Used for the Development of eNFs as Wound Dressings

Polysaccharides represent an indispensable source of versatile materials perceived to be superior to other polymers, due to their beneficial properties such as: Homogeneity, bio-adhesion, and bio-activity [76]. Biopolymer nanofibers such as polysaccharides fabricated via facile electrospinning technique have a series of advantages such as: Ease of processing, excellent biocompatibility, high degree of biodegradability and non-toxicity, and even an antimicrobial action as in the case of chitosan [16]. Regarding the immunogenicity of the polysaccharides used, alginates are considered non-immunogenic even though some researches suggested a correlation between the immunogenic behavior and the high _D_- mannuronate content [6], while xanthan gum exhibits intrinsic immunogenic ability [77]. For chitosan it was reported by Li. et al. that 30% deacetylated chitin is responsible of the activation of macrophages in vivo, inducing the cytotoxic macrophages most effectively [78]. Recent papers have revealed the relationship between the molecular weight of the HA and the immune-adjuvants properties. So, HA with low molecular weight (800–3200 Da) is capable to activate immune-competent cells such as macrophages, whereas the high molecular weight HA (10^7^ Da) is an omnipresent ECM component. The activation of the immune system is mediated by the HA linkage with CD44, CD168, and Toll-like receptors (TLR−2 and TLR-4), specific receptors associated with the host defense against bacterial infection [79].

**Figure 5:**
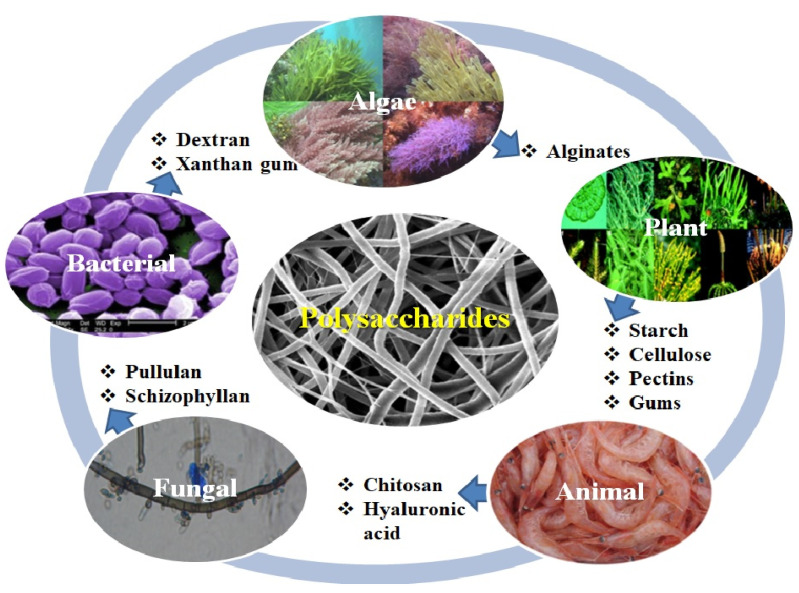
Representation of polysaccharides used for the development of electrospun nanofibers (eNFs) as wound dressings.

The devices formulated from biopolymeric nanofibers may allow a 3D architecture with interlink pores, similar to the ECM, auspicious for tissue regeneration [80]. Several key biopolymeric macromolecules derived from polysaccharides have been stated for enhanced performance in wound healing when used for the formulation of eNFs as wound dressings (Figure 5). Furthermore, since most of the natural polymers are quite difficult to electrospin, many studies revealed the use of composites or blends of these biopolymers with synthetic materials in order to achieve adequate biodegradation rate and proper mechanical properties [80].

A major challenge in the field of treatment of wounds with various etiologies, which is based on electrospun nanofibers mats, is the transfer from laboratory research to clinical research [81]. In order to make this transfer, the scientist’s attention must be directed to overcome some limitations of the current wound dressing formulation such as generation of cell seeded multilayered patches and fine-tuning degradation. For speeding up the journey of polysaccharides eNFs wound healing patches from the bench to the bedside, upcoming advancement and progress should remedy the critical deficiencies and provide more significance on exhaustive and precise clinical applications. A plausible solution for the development of completely functional nanoscale engineered wound healing patches is to create a multidisciplinary team, where scientists with different specialties work in unison, together with the support from regulatory bodies [19].

In what follows, a comparative analysis in terms of mechanical and degradation properties of the eNFs wound dressings based on polysaccharide will be depicted in Table 1.

#### 3.2.1. Algae Origin Polysaccharides

Polysaccharides are a type of bio-macromolecules that exist as cell wall structuring constituents of marine algae. Polysaccharides derived from marine algae are usually connected with pharmacological actions such as immune-modulatory, antioxidant, anticoagulant, and antitumor. The existence of a correlation between the polysaccharides bio-effects and their chemical features has been proven, such as: ratios of mono-saccharides constituent, molecular sizes, types, and properties of glycosidic bonds [109]. Biomaterials based on polysaccharides from seaweed, of which an important place is occupied by alginates, have gained much attention in the domain of wound healing applications, due to the fact that they are abundant, cost-effective, and very versatile [110].

#### 3.2.2. Alginates

**Figure 6:**
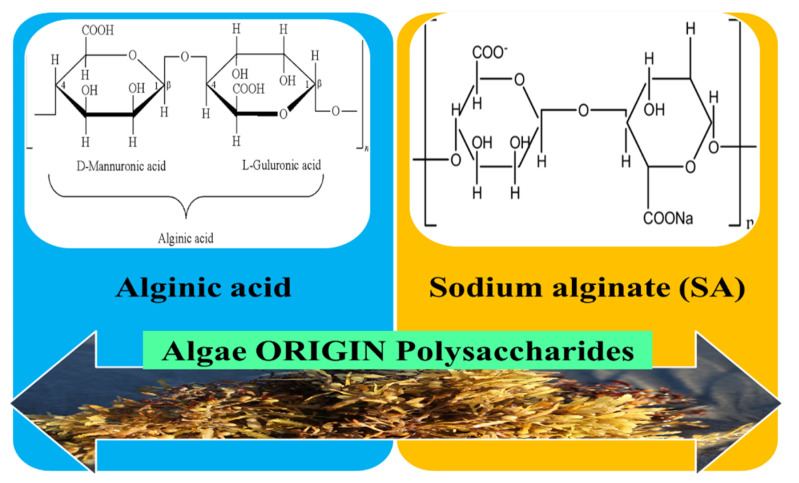
Representation of molecular structure for algae origin polysaccharides for eNFs development used as wound dressings.

Algin or alginic acid is a polysaccharide extensively distributed in the cell walls of brown seaweed (algae) and with which metals such as sodium and calcium forms its salts, known as alginates. Alginate is a biodegradable polysaccharide and a negatively charged polymer originating from brown seaweed or metabolic products of *Pseudomonas spp bacterias* and *Azotobacter vinelandii* [82]. Structurally, it includes two steric different repeating units: α-l-glucuronic acid (G) and β-mannuronic acid (M) 1, 4 linked in varying proportions [83,111]. The negative charge derives from the carboxyl groups placed on the ring scaffold of both G and M monomers (Figure 6) and as a result of the stability of alginate and pH sensitivity the formulation of sustained/controlled drug delivery systems based on alginates have been reported. The unique properties of sodium alginate (SA) such as its good tissue compatibility, non-toxicity, biodegradability, hydrophilicity, and low cost confer the capacity to be suitable for the use in the tissue engineering field, namely skin regeneration and in the curing of exuding wounds with an enhanced healing process [112]. In addition, due to its high hydrophilicity, alginate at the wound area could adequately absorb the surplus of exudate and could also supply a humid medium required for rapid healing [113].

In order to obtain an improved healing action, alginate dressings are taken into consideration as carriers for different bioactive agents, including metal nanoparticles, antibiotics [114], wound healing agents [85], as well as biomolecule and gene delivery systems [115]. Reports demonstrated that the functional characteristics of alginate are substantially increased by blending with other different biopolymers such as silk fibroin, collagen, and chitosan [116]. The advantages of using alginate in combination with other polymers in wound dressing are considerable, such as the wettability’s improvement, the reduction of fibers’ stiffness and also the increase of swelling capacity and adhesiveness. Moreover, in vivo studies demonstrated that wound healing with antibiotic drugs-loaded mats based on SA takes place more rapidly and with a lower risk of superinfection than with drugless scaffolds [84].

Regarding the electrospinning process of alginate, it has been reported that pure alginate is difficult to electrospin due to a series of factors such as the start of alginate gelation at very low concentrations [117], due to the high surface tension, high electrical conductivity [118], and, also because of the absence of chain entanglements from its aqueous solution. Thereby, although alginate has antiseptic properties, can supply a moist medium, has suitable vapor transmission, sufficient water absorptivity, and can absorb the surplus of exudate, it is problematic to develop alginate nanofibers as wound dressing materials via electrospinning pure alginate. A way to surmount this limitation is to associate the polysaccharide–alginate with compatible polymers [119]. Consequently, synthetic polymers such as polyethylene oxide (PEO) or polyvinyl alcohol (PVA) were blended in order to enhance the electrospinnability as well as the mechanical potency of alginate, meanwhile PVA has proven to be a favorable wound dressing material [85]. In recent years, the approach in the field of tissue engineering applications that gained a lot of popularity is represented by nanofiber-reinforced hydrogels due to their analogy to different tissue structures (ECM), improving cell–matrix interactions and enhancing the mechanical characteristics of hydrogels [116]. Recent studies performed on alginates/sodium alginate (SA)-based wound dressings are summarized in Table 2.

### 3.3. Plant Origin Polysaccharides

**Figure 7:**
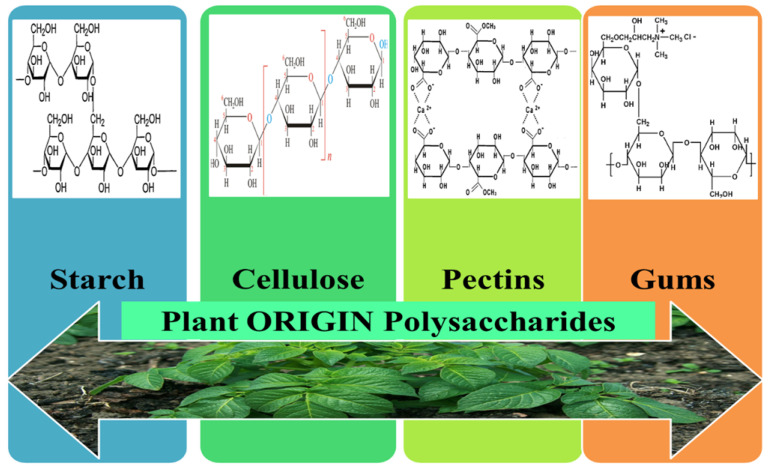
Chemical structures of plant origin polysaccharides that can be electrospun to form eNFs used in tissue engineering as wound dressings.

Polysaccharides isolated from plants are natural polymers located mainly in the plants cell walls and represent the biggest percentage of all biomass. They are constituted of a diversity of monosaccharides, with different structures, and compared to other biopolymers their high number of reacting functional groups gives exquisite versatility. The shared characteristic of plant polysaccharides is their steady structure caused by their very powerful intermolecular interconnections, making them hard to be misshaped by the temperature or by pH shifts. In addition, they are biodegradable polymers with a diversity of biological, physical, or chemical features and forceful hydrophilicity/viscosity that can modify the rheological characteristics of the fluid system [123]. Plant originating polysaccharides have attracted the scientist’s interest by virtue of their biological characteristics such as anticoagulant, antioxidant, and anti-diabetic but also due to their important features such as biodegradation, non-toxicity, and compatibility with environment [124]. In terms of resources, plants are viewed as one of the most substantial sources of polysaccharides. Moreover, superior biological attributes and low processing cost result in them being appropriate for use in wound healing management. The chemical structures for the main plant origin polysaccharide-based eNFs used as wound dressings are represented in Figure 7.

#### 3.3.1. Starch

Starch (S) is one of the extremely abounding biopolymers on earth and a polysaccharide that is outstanding in the research fields of drug delivery and tissue engineering because of its biological characteristics useful for the formulation of wound dressings. Amylopectin (70–80%) and amylose (20–30%) are the predominant chemical constituents of this biopolymer and they can be physically or chemically modified to reach the proper utilization in wound dressing formulation [125].

Starch electrospun nanofibers (S-eNFs) have considerable specific surface area, elevated porosity, and exert biodegradable, biocompatible, and bio-absorbable properties. Consequently, S-eNFs have significant potential in pharmaceutical applications, comprising wound dressing and tissue engineering [126]. The research conducted in the field of starch electrospinning show a difficult process, where handling this material alone does not lead to appropriate mechanical attributes. This is the reason that starch is associated with a series of other biopolymers or synthetic polymers for the development of proper and significantly better wound dressing scaffolds, as it will be further discussed.

Wang and Ziegler report a green technique to develop pure starch-based nanofibers using a wet-electrospinning process. In this process, the use of sodium palmitate for increasing the stability in water of amylose at room temperature has been indicated, as well as for heightening the conductivity of the electrospinning process, and the use of pullulan was also pointed out for stimulating molecular entanglement without the appearance of gelation [87].

Based on the beneficial effects of the association of starch with other polysaccharides, scientists have focused on the fabrication of cross-linked electrospun Starch/Chitosan/PVA nanofibrous mats (S/CS/PVA eNFs) for wound dressing development. The process of cross-linking performed to the uniform prepared bead-free nanofibrous mats will lead to an increased water resistance and to an optimized biodegradation rate. The balanced water absorption and water vapor transmission degree along with the proper porosity of the S/CS/PVA eNFs indicated their capacity in provisioning a moist environment for the wound, suitable for wound breathing, and capable of absorbing the injury’s exudates. The mechanical characteristics in both dry and wet forms validate the capacity to uphold wound site against the outward factors during the healing process, while the antibacterial assays demonstrated good antibacterial potential toward both gram-positive and gram-negative bacteria strains. Moreover, in vitro cytotoxicity was carried out by MTT assay, where appropriate cyto-compatibility and cell viability were revealed, confirming the excellent potential of the tested S/CS/PVA eNFs for wound dressing applications [127].

In another recent study from 2020, a coaxial electrospinning process has been described for the formulation of a core-shell starch-hyaluronic acid (HA)/polyurethane (PU) based eNFs patch, where the S and HA were arranged on the outside part, conferring surface hydrophilicity, biocompatibility, and biodegradability while PU was in the core of nanofiber arrangement improving the mechanical durability [88].

Fonseca et al. explored the formulation of anionic corn starch ultrafine nanofibers with distinct amylose concentrations that showed various morphologies and an average diameter ranging from 70–264 nm. The research indicated that the addition of carvacrol (major constituent of thyme or oregano oils) improve the electrospinning process and also the nanofibers morphologies. On the other hand, it was revealed that the incorporation of carvacrol led to the increase of both the antioxidant and antibacterial activity of the nanofibers against the four pathogen strains tested. Thereby, the eNFs with 30% (*v*/*v*) of carvacrol decreased the growth of *S. aureus*, *S. typhimurium*, *L. monocytogenes,* and *E. coli* by 49.0%, 68.0%, 89.0%, and 62.0%, respectively. The results presented for the formulated S-eNFs embedded with carvacrol point to their potential use as wound dressings [86].

#### 3.3.2. Cellulose

Cellulose, a plant origin polysaccharide, it is one of the utmost naturally abounding and widely used renewable material thanks to its multiple intrinsic properties like biodegradability/biocompatibility, great chemical resistance, and thermal stability. Cellulose has been chosen for the development of 3-D scaffolds, which can provide good aid for growth and cell adhesion. The biomedical applications of mats based on cellulose as scaffolds include repairing, regenerating, and reconstructing almost all type of mammalian tissues. In cell delivery and tissue engineering, cellulose can support the wounds covering and the drug release into the wound site through the post-operative adhesions and hemostasis inhibition [128].

Nano-scale cellulose fibers produced by means of electrospinning (C-eNFs) have significantly attracted the interest of scientists thanks to its correspondingly wider surface area, which confers more surface atoms as compared to its micro-scale [90]. For the development of different electrospun nanofibrous scaffolds used as wound dressings, many types and derivatives of cellulose have been reported, such as alpha cellulose [129], cellulose acetate (CA) [90], ethyl cellulose (EC) [130], and carboxymethyl cellulose (CMC) [131]. Also, like the other polysaccharides, cellulose derivatives can be used in combination with other synthetic polymers or biopolymers for the increase of the electrospinnability, as it can be seen in Table 3.

Yazdanbakhsh et al. describes the formulation of C-eNFs that incorporate a fluoroquinolone antibiotic, ciprofloxacin hydrochloride (Cip). In this study, the use of α-cellulose extracted from wheat bran has been reported, obtained with the help of trifluoroacetic acid (TFA)/methylene chloride (MC) as a mixed solvent. The alpha-cellulose eNFs impregnated with Cip showed a higher inhibitory activity on *S. aureus* ATCC-25933 compared to the standard disk. Regarding the results of this study, wound scaffolds based on wheat bran derived α-cellulose eNFs are efficient in wound healing as a result of proper porosity and morphology, which confers permeability towards humidity and oxygen, ease of application, and no adhesion to wound. Also, it was demonstrated that drug-loaded α-cellulose nanofibers can decrease the wound size as it has optimal drug-release properties, in comparison with α-cellulose nanofibers without other incorporated drugs [129].

Another recent study that is based on the embedment of ciprofloxacin into nanofibrous mats derived from cellulose has been led by Li *et al.*, where ethyl cellulose (EC) is combined with another polymer polyvinylpyrrolidone (PVP). Both polymers are low-cost, biocompatible, and electrospinnable, whereby PVP is a hydrophilic polymer, while EC is a hydrophobic and inert polymer appropriate for continuous release systems. In vitro drug release assays were performed in order to mimic drug release into the wound area from the formulations and it was indicated that the hydrophilic nanofibers displayed a much more accelerated release than their hydrophobic equivalents. The ciprofloxacine mechanism of release was characterized by a combination of drug diffusion and polymer erosion, and the EC-eNFs indicated a close to zero-order Cip delivery over a period of three days. Regarding the cytotoxicity, it was revealed that the fibroblast cells were able to grow and proliferate on the studied nanofibers. Also, inhibition zone tests showed that the replication of both gram-negative and gram-positive bacteria is productively inhibited as a consequence of the presence of Cip in the eNFs. Due to insignificant discrepancy between the fibers collected on gauze and on foil it was concluded that electrospinning can be effectuated directly onto a gauze substrate for smart fabric preparation [130].

#### 3.3.3. Pectins

Pectin polysaccharides (pectins) are a complex and dynamic family of polysaccharides characterized by an irregular structure of carbohydrate chains [92]. Pectin, along with chitosan and alginate, are the most extensively utilized ionic polysaccharides in the field of wound dressing development [135]. The different molecular structures of these pectins result in various characteristics of their micro/nano-shaped dressings, which fit in various biomedical applications. Pectin is a linear, heterogeneous polysaccharide isolated from apple pomace and citrus fruit peels and is mainly composed of D-galacturonic acid (GalA) units, partially methoxylated and attached in chains by (1–4) glycosidic bonds with alternating side chains of a (1–4) D-galactose and D-arabinose [136]. Pectic acid represents the acid form of pectin and is able to convert into a salt called pectinate after reacting with a base. Having the advantage of moderate hydrophilicity, pectins can act as exudate-absorbing constituents in hydro-colloidal wound dressings [137].

In a comparison experiment led by Chen *et al.*, the properties of the mats of alginate (Alg), pectinate (PCT), and chitosan (CS) developed as eNFs using the co-polymer polyethylene oxide (PEO were analyzed). For the formulation of the PCT–eNFs, the 6.5% aqueous solution of sodium pectinate was blended with a 5% PEO solution in a mass ratio of 80/20 (PCT/PEO), adding DMSO as co-solvent. The final PCT/PEO electrospinning solution had a pH value of 7, was fed into a 5 mL syringe with an 8-gaze stainless steel needle, and a voltage of 8–18 kV between the syringe tip and a grounded flat collector found at a distance of 15–20 cm was applied. In spite of the fact that all three biopolymeric nanofiber scaffolds had similar vapour permeability and mechanical strength it was revealed that the PCT-eNFs could absorb, within less time, 3.6 times and 1.2 times more exudate compared to CS-eNFs and Alg-eNFs, respectively. Moreover, the PCT-eNFs revealed much more elevated antibacterial activity (73.1%) than the CS-eNFs and Alg-eNFs (17.1% and 11.8%, respectively). All these findings imply that the PCT-eNFs scaffold could act as a superior wound dressing in comparison with the chitosan and alginate nanofiber patches [137].

In 2018, a research group investigated the preparation of PCT-eNFs by an intial oxidation of pectin with periodate in order to form aldehyde groups capable of cross-linking with adipic acid dihydrazide (AAD) for a covalent connection between pectin macromolecular structure with AAD linkers. It was found that in comparison with standard Ca^2+^- cross-linked PCT-eNFs, the pectin nanofibers obtained by prior oxidation followed by cross-linking with AAD revealed higher cell adhesion ability. Additionally, the oxidized/cross-linked NFs exhibited high biodegradability (complete degradation within three weeks) and excellent mechanical strength. Combining all the data and all the results obtained confirms that the PCT-eNFs formulated by prior oxidation and cross-linking with AAD are auspicious candidates for in vivo applications comprising tissue engineering and wound healing [91].

#### 3.3.4. Gums

Gums, a broad group of polysaccharides, are used as a new source of biopolymers for the eNFs formulation [138] with different pharmaceutical applications. Several factors that can limit the process of gum electrospinning have been listed in the specialized literature, such as elaborated structural conformation, concentration, solubility, viscosity, conductivity, surface tension, vapor pressure, molecular chain entanglement, and gelling properties [61]. A correlation between the solvent nature (organic or aqueous) and the gums’ electrospinnability as well between the gums’ molecular characterization and their fractionalization procedure has been demonstrated, resulting in high or low molecular weight fractions. Furthermore, other critical factors that cause a polymeric solution to be subjected to the process of ellectrospinning in order to form eNFs are the molecular interactions: Polymer–polymer conjunction, polymer-small molecules (nanoparticles, additives, or salts, etc.), and supramolecular polymers-small molecules [139]. The process of electrospinning gums with high molecular weight is demanding as a consequence of their heterogeneity, polydispersity, and abounding functional groups such as –NH_2_, –CO, –OH, –COOH, etc. In the case of not preserving all of the key parameters needed for the electrospinning process, instead of eNFs formation, only droplets formation was reported [94].

Amongst the polyvalent group of carbohydrate polymer gums with plant origin, we will further discuss the ones used for wound dressing based on eNFs scaffolds: Gum guar (GG) [140], gum Arabic (GA) [95], Gum Azivash (GAz) [97], gum karaya (GK) [93], and gum tragacanth (GT) [141].

Guar gum (GG) is a cluster bean derived from the drought leguminous crop*-Cyamopsis tetragonoloba* L. GG has galacto-mannan chains of (1→4)-linked-β-D-manno-pyranosyl units connected by (1→6) linkages with single α-D-galacto-pyranosyl units [142]. Gum Arabic (GA) also known as acacia gum or meska it has its source from *Acacia seyal* and *Acacia senegal.* The main constituents of AG are both galactose and arabinose, monosaccharide sugars, glucuronic acid (sugar acid derived from glucose), and rhamnose (deoxy sugar). Azivash or *Corchorus olitorius L.* is a medicinal and edible plant found in the tropical countries of Africa and Asia. The leaf gum of Azivash is non-toxic with a high molecular weight about 940 kDa. Gum Azivash (GAz) is capable of jet formation and not fibers formation, this being the reason that it will be associated with other polymers for proper eNFs formulation. It is reported that the hydrocolloid viscosity of Azivash at 0.5% (*w*/*w*) concentrations is higher than hydrocolloidal viscosity of agar gum [97]. Gum karaya (GK) derived from *Sterculia urens* is a partially acetylated gum with a high molecular mass of 16 × 10^9^ Da. GK it is composed by neutral sugars such as arabinose, galactose, rhamnose, and acidic sugar fractions of uronic acids (glucuronic and galacturonic), which demonstrates its utilization as biosorbent [143]. Gum tragacanth (GT) represents one of the most vastly exploited natural gums, which has established applications in wound management due to its attractive properties such as non-toxic nature, biodegradability, long shelf-life features, and greater resistance against microbial aggressions [144]. Gum Tragacanth (GT) is a component of *Astragalus*, a genus of approximately 3000 species of herbs, pertaining to the legume family of *Fabaceae*. GT comprises two constituents: Tragacanthic acid or bassorin, a water-insoluble component capable to swell and form a gel and tragacanthin, a water-soluble fraction formed by a nucleus of α-(1–4) galactose rests with branched section of arabinose. The water-swellable, highly branched component is represented by tragacanthic acid, which is composed of linear strands of α-(1–4) galacturonic acid with fractions of fucose, galactose, and xylose [94].

An overview on representative eNFs from gums with plant origin and their properties and applications as wound bio-degradable dressings is depicted in Table 4.

### 3.4. Animal Origin Polysaccharides

**Figure 8:**
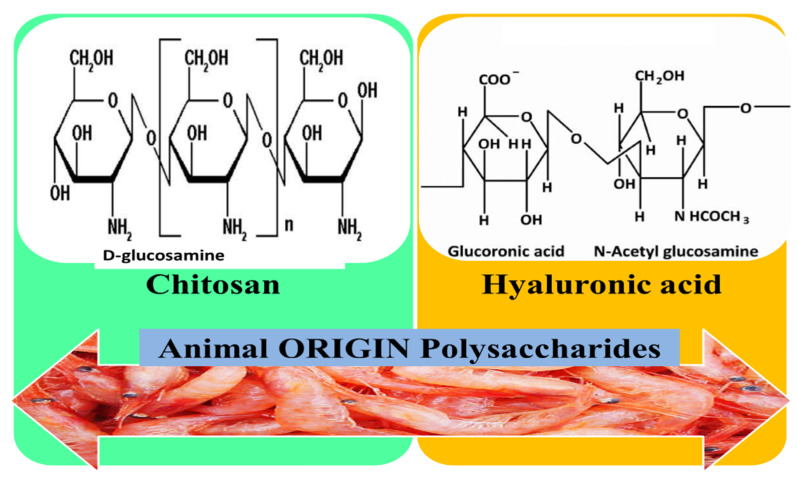
Chemical structures of animal origin polysaccharides used for the development of wound dressings based on eNFs mats.

Animal polysaccharides mainly include hyaluronic acid and chitosan (Figure 8), which have proven antioxidant, antibacterial, anti-inflammatory, and other biological properties, so they can be used in drug development and different biomedical fields, including wound healing management [147].

#### 3.4.1. Chitosan (CS)

Chitosan is the partially deacetylated derivate of chitin, which is the second most abounding naturally occurring polysaccharide, after cellulose, and which consists of arbitrarily scattered units of N-acetyl-d-glucosamine and d-glucosamine β-linked [6]. CS is a natural compound that has proven its key role in wound healing due to its proper properties: Good interaction with molecules from the phospholipid membrane, increasing the analgesic and hemostatic effect, accelerating the proliferation of fibroblast cells, stimulating neutrophils and IgM, and enhancing the activation of macrophages and the production of ECM [1]. To these beneficial characteristics in the wound healing process is added its antimicrobial activity owing to the cationic nature of CS, which can determine the interaction between the negatively charged functional moieties situated on the surface of the bacteria’s cell wall and the –NH_3_^+^ group. This interaction between differently charged groups can modify the bacterial surface morphology, which either can augment the membrane permeability, causing release of intracellular substances (nucleic acids, glucose, and proteins like lactate dehydrogenase), or it can diminish membrane permeability, hampering the nutrient transport [148].

Being a polyelectrolyte in acidic medium, CS has proven challenging to electrospin, but in spite of that, many studies revealed the common method to enhance the electrospinnability of CS by blending it with other easily electrospinnable polymers like polyvinyl alcohol (PVA), polylactic acid (PLA), and polyethylene oxide (PEO) [74,149]. Thus, due to its cationic charge, several studies show that the mixture of polymers, including other biopolymers, will improve the formation of nanofibers and also will enhance the functional characteristics, as depicted in Table 5.

#### 3.4.2. Hyaluronic Acid

Hyaluronan or hyaluronic acid (HA) is a naturally occurring linear and non-sulfated glycosaminoglycan composed of N-acetyl-d-glucosamine and d-glucuronic acid and represents a major constituent of the ECM, connective tissue, and cartilage. Hyaluronic acid creates a viscous matrix in the ECM within which elastin and collagen fibers are incorporated [6]. Due to its good biodegradability, biocompatibility, high degree of wettability, non-immunogenic character, and ability to be chemically modified, HA has important bio-applications in the fields of wound healing, tissue engineering, drug delivery, and visco-supplementation [153].

HA is vastly used for wound healing applications owing to its strong potency in terms of elevated potential to raise the water absorption ability, which impedes the desiccation of injured tissue surface and which supplies a moist milieu, therefore promoting healing process. At the wound site, HA produces the increase of collagen secretion by fibroblast/keratinocytes proliferation and also promotes the differentiation of fibroblast into myo-fibroblasts [88]. Being the principal component of the skin’s ECM, HA provides vital contribution to the wound healing process, by the production and release of pro-inflammatory cytokines and interleukins and by promoting the development of a fibrin clot. Additionally, it reduces the inflammatory cells infiltration with re-epithelization and granulation improvement, and it enhances the formation of blood vessels as well, that are of extreme relevance for skin regeneration’s melioration [101]. HA has been elected by scientists for the formulation of different electrospun mats as wound dressings, since it exhibits strong-water retention ability, biodegradability/biocompatibility, and advantageous actions on wound healing process.

However, electrospinning pure solutions of HA is highly challenging owing to its strong surface tension, and to its chain rigidity that derives from the intra-molecular hydrogen bonds and from the long-electrostatic interactions, which will lead to the viscosity increase without favoring chain entanglements. Therefore, in order to diminish the surface tension and viscosity, HA has been electrospun at 40 °C in the presence of DMF [154] or at high pH aqueous ammonium solutions [155]. The formation of nanofibers directly from pure HA aqueous solutions using an electro-blowing technique merging air flow, electrospinning, and heating has been mentioned [156]. Alternatively, for obtaining regular nanofibers, researchers have been associating HA either with bioactive agents or natural polymers (for the increase of HA’s biological performance) or with synthetic polymers (for the enhancement of its electrospinnability and mechanical properties) [101,157].

One direction that arouses the interest of scientists is the concept of bi-layered scaffold developed to imitate the genuine characteristics of native skin. The double-layered mats have the following main advantages: maintaining an adequate level of hydration in the wound site for proper cell incorporation and mechanical retention of the scaffold. Bilayered eNFs scaffolds based on CS and HA blended with synthetic or natural polymers were used for wound healing as shown in earlier papers [158,159]. Another direction of the research consists of the inclusion of metallic nanoparticles (silver) to formulate a wound dressing nanofiber scaffold, containing biologically adsorbent materials. For this formulation, the silver nanoparticles will act as an anti-inflammatory and antioxidant that defend the cells from the devastating effect of raised amounts of reactive oxygen species (ROS) and in the same time, will facilitate wound healing process [153]. Also, recent researches in this field with the formulation of eNFs with application in wound management are highlighted in Table 6.

### 3.5. Fungal Origin Polysaccharides

**Figure 9:**
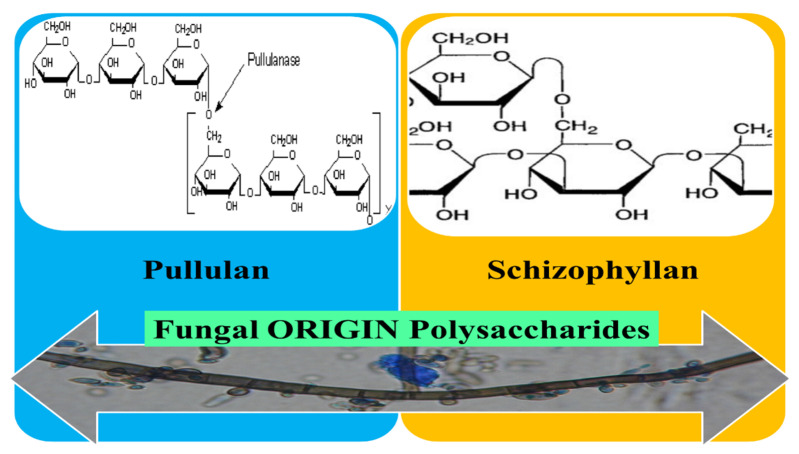
Chemical structures of fungal origin polysaccharides used for the formulation of potential material dressings with nanofibrous structure.

Fungal polysaccharides are synthesized by many species of fungi and this review focused on the pullulan and schizophyllan, capable to form eNFs (Figure 9). In accordance with their bioactive characteristics like antioxidant, anti-bacterial, and immune-modulating, fungal polysaccharides are being explored for numerous health-care applications, including tissue engineering and preparation of wound dressing materials [161].

#### 3.5.1. Pullulan

Pullulan (PUL) is an extracellular microbial polysaccharide generated by the fungus-like yeast, *Aureobasidium pullulans* **[162]**. Structurally, PUL consists mostly of repeating malto-triose units connected by α (1,4) and α (1,6) glycosidic bonds in a ratio of 2 to 1 [163] and it has been selected for the preparation of ultrathin electrospun nanofibers [164]. PUL is non-mutagenic, non-toxic, tasteless, and odorless and due to these characteristics, it has widespread use for different pharmaceutical and biomedical purposes [165]. In spite of its biocompatibility, skin tissue engineering applications of PUL are hindered by its high hydrophilicity, which limits the support for cellular attachment and proliferation, and which prevents the adsorption of proteins. In order to overcome these limitations, composite scaffolds were formulated, scaffolds that incorporate inorganic materials, tissue-specific growth factors, and ECM-proteins [166].

For improving the PUL’s electrospinnability it was demonstrated that the association with protein solutions is very useful due to the shaping of hydrogen bond between PUL and proteins by transforming the characteristics of the polymer solutions [167]. Therefore, many studies have concentrated on the use of proteins and PUL blends that are reciprocally compatible for eNFs formation [104,168]. Table 7 aims to present the latest discoveries in the field of nanofibers derived of pullulan used as potential wound dressing materials. 

#### 3.5.2. Schizophyllan

Schizophyllan (SPG) is a non-ionic, water-soluble exo-polysaccharide originating from the wood-rotting filamentous basidiomycete fungus*-Schizophyllum commune* [170]. SPG belonging to the homoglucan family is made up of the major β (1–3) glucan sequence linked via β (1–6) glucan bondage to every third entity from polysaccharide’s structure [171,172]. It was mentioned that during the renaturation process, SPG has the ability to form a one-dimensional hydrophobic hollow within the helical super-structure of SPG, therefore can accept nanoparticles, molecular constituents, functional polymers to form water-soluble unidimensional nano-composites, whereas individual molecular assemblies and conjugated polymers can be embedded into the one-dimensional cavity [173]. SPG has been examined and used for the formulation of diverse nanocomposites: SPG-nanoparticles [174], SPG-nanogels [170], and SPG-double-network antibacterial hydrogel [172] in different biomedical applications.

A recent examination performed by Safaee-Ardakani et al. reports the formulation of eNFs from a blend solution composed by 1.5 *w/v*% aqueous SPG solution mingled with a 10 *w/v*% aqueous solution of polyvinyl alcohol (PVA) at different volume ratios, where a dependable linear liaison was constituted between the fiber diameter and solution characteristics. The role of adding PVA was for the enhancement of SPG’s electrospinnability, due to their functional groups capability to react with SPG, while SPG acts for improving the immune system via activating macrophage cells. Also, for improving the mechanical attributes of eNFs mats, a vapor cross-linking process with glutaraldehyde was performed, resulting in bead-free, smooth, and contiguous nanofibers. The formulated SPG/PVA eNFs were further evaluated in terms of indirect cytotoxicity with mouse fibroblasts (L929), when it was showed a high efficiency in improving cell adhesion and proliferation. The biological evaluation proved that the nanofibrous mats exhibited a lack of cytotoxicity to the growth of L929 cells combined with an excellent in vitro biocompatibility. The study concluded that the SPG/PVA eNFs scaffold obtained in a volume ratio of 20:80 proved to be a convenient matrix for enhancing the wound healing, as it could improve migration and cell proliferation, so the eNFs have the capability of being processed as scaffolds for either skin recovery or wound dressing [60].

### 3.6. Bacterial Origin Polysaccharides

**Figure 10:**
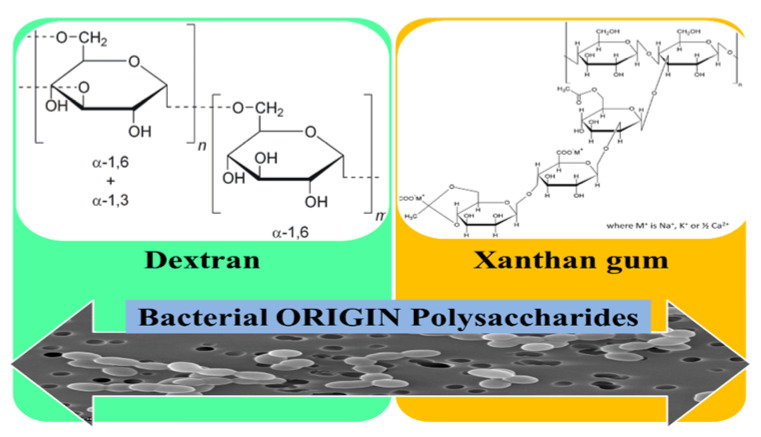
Molecular structures for bacterial origin polysaccharides used for eNFs formulation as wound dressings.

Due to their high growth rates of microorganisms, to the possibility of enhancing productivity as well as customizing the biopolymers’ desirable properties by modifying the bioprocess conditions, the bacterial origin polysaccharides are of special interest in the scientific community [175]. Microbial polysaccharides are mainly exo-polysaccharides that bacteria secrete for their own purposes henceforth they do not provoke a biological response from cells, so they do not biologically interact with human tissues. To overcome this drawback, scientists have embedded bioactive substances or inorganic materials into the scaffolds based on bacterial polysaccharides via physical and chemical strategies [176]. Electrospun nanofibers (eNFs) obtained from bacterial biodegradable polymers have attracted a privileged attention for the wound healing field as they are non-antigenic, histo-compatible, and readily washed from the wound area [177]. The two most widely used bacterial polysaccharides for formulation of wound dressings are dextran and xanthan gum (Figure 10).

#### 3.6.1. Dextran

Dextran (DXT) is a neutral polymer derived from *Streptococcus mutans* and *Leuconostoc mesenteroides*, lactic acid-producing bacteria and is composed of α-(1→6) and α-(1→4) glucopyranosyl linkages. Louis Pasteur was the one who initially discovered dextran, as a fermentation by-product of wine [178]. The bacterial polysaccharide DXT is a readily available and water-soluble biopolymer that exhibits good biodegradability and biocompatibility and that had been used in various medical applications, including skin tissue repairing [106]. Most importantly, DXT it is not only water-soluble, but also it can be dissolved in different organic solvents. This exclusive solubility feature of dextran makes possible the direct association with hydrophobic polymers like polyurethane (PU) for nanofibrous mats’ formulation via electrospinning. The hydrophilic polymers have high cell affinities, but exhibit low mechanical strength, while the biodegradable hydrophobic polymers generally have inverse properties such as high mechanical strength but with an absence of cell affinity. Therefore, blending hydrophilic and hydrophobic biodegradable polymers has the ability to overtake the deficiency of the individual materials’ characteristics [179,180]. So, DXT being a versatile bio-macromolecule can be manipulated for the formulation of eNFs by blending with either hydrophobic biodegradable polymers or water-soluble bioactive agents for skin tissue regeneration applications.

Due to the DXT’s hydrophilic character, the process of cross-linking is imperative for tailoring its biodegradation stability as well as for retaining its mechanical features in moistened conditions. Thereby, DXT solubility’s in water must be exceeded by introduction of inter-molecular connections via the use of different cross-linkers. Even though standard cross-linking ways attend to use toxic substances, for example glutaraldehyde, it has been reported that simple mixing dextran with boric acid (BA), in watery solutions, can be electrospun for the preparation of DXT-BA-eNFs with controlled degradation times. This led to the formation of a steady network capable of hindering the drug release time up to 500% when compared to pure DXT-eNFs. Also, it was found that the presence of boron in the nanofibers nucleus proven by combining FT-IR, X-ray photoelectron spectroscopy (XPS) and thermo-gravimetric assays indicates a gradual surface degradation discharge. The study concluded that by optimizing boron concentration in multi-layer wound patches processed as DXT-based eNFs, the drug delivery could be methodically administered and controlled to the targeted site [107]. Recent reports of DXT-based eNFs development for skin tissue regeneration are presented in Table 8.

#### 3.6.2. Xanthan Gum

Xanthan gum (XG) is an anionic extracellular polysaccharide, which is secreted by the *Xanthomonas campestris* bacterium and used for the generation of self-assembled micro or nano-scales structures with prospect use in controlled drug delivery, regenerative medicine, and tissue engineering. It is the second bacterial polysaccharide, after dextran, to be industrially commercialized and due to its properties of non-sensitizing, non-toxic, and owing to the lack of skin irritation, it was authorized by the Food and Drug Administration (FDA) in 1969. XG’s primary structure was established for the first time in 1975 and consists of α (1→4)-linked glucose units substituted at O-3 with a tri-saccharide formed by one glucuronic acid fraction between two mannose parts comprising pyruvate remnants and acetyl group [77,183]. The secondary structure of XG can be formed at low salt concentrations or high temperatures and can be depicted as a five-fold right-hand helical arrangement with a diameter of 1.9 nm and a pitch of 4.7 nm, capable of thermally induced configurational conversion [184]. XG is stable in a wide span of ionic strength, pH, and temperature and is also soluble both in hot and cold water, requiring vigorous shaking when exposed to aqueous environment to prevent the chump appearance [185]. The solutions of XG comport as non-Newtonian fluids and have a greatly pseudoplastic behavior, where the apparent viscosity altered considerably with the shear rate and/or with time [186]. In general, the thermal stability of XG over hydrolysis is superior to many other hydro-soluble polysaccharides or biopolymers, presumptive because of the XG’s ordered helical design that safeguards the molecules from de-polymerization [77,187]. The polyelectrolytes with high molecular weight contribute to better formation of nanofibers thanks to their capacity to create a more viscous assembly that settles the interface. The broader area instituted can facilitate complexation and support interactions with other biological agents [188].

The main challenges of subjecting XG to electrospinning process are thixotropic behavior, deficient gelling ability in an aqueous medium, and insufficient chain entanglement. However, it was reported that when using formic acid as an electrospinning solvent without co-polymer addition, the rheological behavior of XG was reversible by shear thinning, which can effortlessly surmount the above-mentioned obstacles leading to the mandatory rheological features [94]. Shekarforoush et al. explored the formulation of pure XG polysaccharide eNFs making use of formic acid as a solvent, when the morphological analysis by SEM illustrated uniform nanofibers with average diameters spanning from 128 ± 36.7 to 240 ± 80.7 nm in relation with the XG concentration (0.5–2.5 wt/vol%). The FT-IR and circular dichroism assays analyze the esterification reaction that takes place between the formic acid and the pyruvic acid fractions of xanthan. Consequently, the esters formed neutralize the pyruvic charges, which will successively stabilize the helical configuration of XG [189].

A recently study performed on the incorporation of curcumin (Cr) into XG-CS polysaccharides eNFs was carried out by Faralli et al., who indicated that when immersed in aqueous HBSS medium, nanofibrous mats remained stable at pH values of 6.5 and 7.4, generally owing to the capacity of oppositely charged XG-CS polyelectrolytes to develop ionically linked eNFs. The research also reported that after 24 h of eNFs’s incubation with Caco-2 cells monolayers, a cell viability of ~80% and an increased in vitro transepithelial permeability of curcumin without jeopardizing cellular viability were exhibited. At the same time, a 3.4-fold growth of curcumin permeation when the polyphenol was incorporated into XG-CS eNFS was found, when compared to the free curcumin, a phenomenon that can be explained by contact interactions between the Caco-2 cells and eNFs, which trigger the opening of the tight junctures [190].

Another research conducted by the same group of scientific researchers focused on the development of stable eNFs from XG-CS viscoelastic solutions for the embedment and release of curcumin (Cr). It was found that adding Cr will diminish the adhesion properties of the nanofibers, due to its hydrophobic characteristics, and it was also shown that the curcumin release was pH-controllable by the pH of the release medium. The research highlighted that the XG-CS eNFs can act as a carrier for the embedment of hydrophobic bioactive substances with elevated incorporation capacity, physical steadiness in aqueous environment, and with long-term pH-controlled delivery properties, in different biomedical applications, including skin tissue regeneration [108,191].

## 4. Conclusions and Perspectives of Research

The wound healing process is reputed as one of the most elaborate phenomena that happens in the human body because it fulfills a key role in the body homeostasis preservation. At the present time, scientists have been developing/formulating/processing various categories of wound dressings including films, sponges, hydrogels, and polymeric mats to enhance and accelerate the healing process. Among them, formulating electrospun nanofibers membranes derived from polysaccharides has been the aim of a large number of scientific investigations as a consequence of the constitutional resemblance with the skin ECM, the elevated surface area-volume ratio, porosity, and ability to perform as a drug delivery system. Furthermore, it was reported that the eNFs membranes also can serve as a barrier for avoiding the appearance of infections as well as can support cell adhesion, differentiation, and proliferation.

One of the limitations of the polysaccharides-based dressings refers to the compatibility between the degradation rate of the eNFs mats and the rate of tissue regeneration. Different studies indicated that with the increase of the degree of degradation of the eNFs scaffold above the optimal level, the mechanical integrity will be diminished, which will lead to the slowing down of the tissue regeneration process. On the other hand, the very low degradation rates of the dressings do not have a favorable effect on rapid and efficient epithelial regeneration. Thus, there is no uniform rate of degradation valid for all types of formulated dressings, but this will depend on the nature and specific properties of the polysaccharide used. For example, in the process of degradation encountered in the electrospun nanofibers based on cellulose acetate and gelatin, fibroblast affinity and elevated collagen secretion were revealed when the tissue remodeling emerged progressively [29].

Another disadvantage of using polysaccharides derivatives-based eNFs in tissue engineering is related to the small intra-nanofiber pore size, corresponding to a 2D environment, which can determine a non-optimal degree of infiltration of the cells in the interior of the scaffolds. For overcoming this obstacle, different attempts have been made to formulate scaffolds with a larger intra-nanofiber pore size to allow the scaffolds to display a 3D environment. Thus, the researchers turn their attention to the development of 3D scaffolds, and a promising method is represented by combining several biopolymers with different properties in terms of solubility, wettability, and flexibility, which will lead to a controlled intra-nanofiber pore size [49].

In this review, a description was made of the four steps of the wound healing process, highlighting the factors that influence this process. Further, a description of the electrospinning process was made in view of electrospun nanofibers formation as wound dressings, with the detailing of the different types of electrospinning that can be effectuated. A preface of each category of polysaccharide (according to their origin) used for the formulation of eNFs as wound dressings followed by a generalized introductory discussion has helped in achieving better insight into the polysaccharide electrospinnig process. With this information, the present review aspires to provide a structured vision of recent techniques on how both organic and inorganic bioactive compounds incorporated into nanofibrous scaffolds can boost polysaccharide eNFs bio-functionality. We have presented a brief overview of the most widely used plant, animal, fungal, and bacterial origin polysaccharides for the formulation of different nano-scaled and smart wound dressings. The eNFs formulation has the main advantage of the possibility of drugs and biological molecules encapsulation, which can be delivered in accordance with the wound healing stage and can improve each particular stage for accelerating wound healing. All the scientific research consulted indicates that electrospun nanofibers-based dressings derived from polysaccharides exhibited more desirable characteristics compared to traditional dressings regarding the cost, preparation, efficient drug delivery, and enhanced wound healing time.

## Figures and Tables

**Table 1:** Comparative analysis in terms of mechanical and degradation properties of the eNFs wound dressings based on polysaccharide.

Polysaccharide	Mechanical Properties/Flexibility/Elasticity	Degradation Properties
I. Algae Origin Polysaccharides		
**Alginates** **(Alg)** **SA** **(sodium alginate)**	- blending with synthetic polymers-PEO or PVA or natural polymers (cellulose nanocrystals, pullulan etc.) can enhance the mechanical potency of alginate [82]; - crosslinking with calcium chloride improves nanofiber hydrophilicity [82]; - human platelet lysate loading of PUL/SA eNFS affected scaffold stiffness by enhancing system deformation and by decreasing their elasticity [83].	- drug release from nanofibers can be realized by penetration from pores, drug desorption from the surface, or by matrix degradation [84]. - when honey is used as a bioactive substance, after Alg/PVA eNFs were immersed in PBS, the honey has dissolved, resulting in the degradation of eNFs and core-structure shattering for water-uptake, thus, diminishing the water absorption capacity [85].
**II. Plant Origin Polysaccharides**		
**Starch (S)**	- the mechanical features of starch eNFs from pure starch are poor but can be improved by the addition of actives molecules such as carvacrol [86] or by association with pullulan, which helps the electrospinning process and ameliorates the fiber morphologies [87].	- the hydroxyl groups in starch structure enhance the water absorption and crosslinking process lowers water uptake and implicitly the degradation rate [88]; - the addition of antioxidants intends to bypass thermal degradation of polymers during manufacturing [86].
**Cellulose** **CA** **(Cellulose acetate)**	- the tensile strength/Young’s modulus values of cellulose eNFs could be increased by heat/chemical treatment, because during heat treatment eNFs gets crosslinked, so reaching at crossover points will lead to the bonding of nanofibers [89].	- in vitro degradation and drug release study revealed that cellulose eNFs are absorbable-degradable barriers, so they are prospective bio-degradable drug release devices [89]. - by adding silver-sulfadiazine (SSD) the thermal degradation of CA nanofibers was increased [90];
**Pectins (PCT)**	- if the PCT eNFs are subjected to prior oxidation or crosslinking reactions such as calcium chloride or adipic acid dihydrazide [91] then an improvement of the mechanical properties has been observed together with a reduction of the release of the incorporated active substances [92].	- the high density of crosslinking will result in a reduction of swelling and elasticity, which can cause the appearance of brittle gels at the moment of degradation for the eNFs derived from PCT [92].
**Gums** **GA (Gum Arabic)** **Iranian Gum Tragacanth (IGT)**	- co-solvent (glycerol) or partner polymers such as PVA, PEO, PCL by increasing the surface tension will facilitate the formation of eNFS derived from different gums [93]; - emulsifiers and thermal post-treatment can contribute to the improvement of mechanical attributes of gums derived eNFs [94];	- GA/PCL eNFs indicated degradation with environmental pH variation by decreasing the pH into the acidic range, beneficial in wound healing [95]. - high percentage of IGT confers superior mechanical, chemical stability, and degradation of IGT/PVA eNFs [96]. - green tea extract (catechin) substantially increased the thermal stability of PVA/Gum azivash eNFs, resulting in an augmented thermal degradation temperature [97].
**III. Animal Origin Polysaccharides**		
**Chitosan (CS)**	- the association with synthetic polymers-PEO, PVA, PCL or biopolymers (gelatin, silk sericin, alginates, hyaluronic acid etc.) can enhance the mechanical characteristics and in the same time can improve considerabily the electrospinning process of chitosan based eNFs [98,99];	- combining synthetic polymer (PVA) with CS for the formation of CS/PVA eNFs incorporating honey as bioactive agent, TGA analysis showed a 2 phase degradation, the first weight-loss corresponding with the PVA degradation (250 °C) and the second one (350 °C) with the chitosan ones [100].
**Hyaluronic acid (HA)**	- similar with CS, many studies demonstrated improved mechanical features of the HA-nanofibers obtained by means of electrospinning when HA it was blend with other biopolymers (collagen, starch, gelatin, chitosan) and synthetic polymers (PVA, PEO, PU, PLGA) [101]; - PCL/HA nanofibers showed down-regulated collagen I expression and an up-regulated collagen III expression together with proper mechanical properties for wound application [102].	- due to the rapid and high in vitro/in vivo degradation of HA a periodic replacement of wound dressing is necessary, which can conduct to the formation of new lesions, enhanced risk of infection and suffering to the patient [101]; - to overcome the above-mentioned drawback the eNFs can incorporate biomolecules such as adhesive proteins (fibrinogen, fibronectin) or antimicrobial agents (natural products, antibiotics, silver nanoparticles) [103].
**IV. Fungal Origin Polysaccharides**		
**Pullulan (PUL)**	- for the formation of PUL eNFs the association with proteins (pea protein) solution together with thermal cross-linking has indicated good mechanical properties [104]; - biopolymer (chitosan, sodium alginate) association with PUL was also reported for the development of eNFs with suitable mechanical attributes for the use as wound dressings [83,105].	- composite eNFs have different degradation comportment than the eNFs formed from pure PUL eNFs owing to the covalent bonds formation by the crosslinking with other biopolymer (chitosan) or biomolecules (tannic acid); - the composite eNFs reveals unstable thermal stability with onset decomposition at approx. 185 °C, lower than the onset degradation temperature of pure PUL eNFs (about 250 °C) [105].
**Schizophyllan (SPG)**	- SPG and PVA blend indicated a tensile strength of nanofibrous mat with great similarity with to the tensile strength of natural skin [60].	- vapor cross-linking process with glutaraldehyde will lead to bead-free SPG eNFs characterized by a degradation rate suitable for wound healing application [60].
**V. Bacterial Origin Polysaccharides**		
**Dextran (DXT)**	- DXT, water-soluble biopolymer with low mechanical strength and by association with other polymers (PVA, PU, PCL), can improve the mechanical properties of DXT eNFs [106].	- cross-linking is imperative for tailoring DXT biodegradation stability, and the simple mixing with boric acid will lead to a gradual surface degradation discharge [107].
**Xanthan Gum (XG)**	- XG rheological and mechanical properties are improved at the addition of formic acid and at the association with CS for XG eNFs formation [108].	- XG treated at high ionic strengths facilitated more mechanical degradation due to the more rigid molecules in ascending order, which showed an effective stress [77].

**Table 2:** Recent studies on sodium alginate-based electrospun nanofibers (SA-eNFs) used as wound dressings.

Biopolymer/ Copolymer	Bioactive Agent	Type of Electrospinning	Main Findings	**References**
**SA/Polyethylene** **oxide (PEO)**	**Gabapentin/Acetamino-phen**	Double-layered Blend electrospinning	- Bi-layered formulation: eNFs loaded with gabapentin, where the contact layer consists of PEO and second layer of SA; acetaminophen was added in the eNFs design for synergizing the analgesic effect; - Drug release from the coating layer exhibited a first-order kinetic model, whereas the release from the second layer a Hixson–Crowell kinetic model; - Potential application for decreasing pain scores with a reduction of side effects in burn patients.	[120]
**SA/PVA**	**-**	Blend electrospinning/three-dimensional (3D) printing	- The double-layered carriers with eNFs as the surface layer proved decreased adhesiveness and increased physical durability compared to the solvent cast (SC) film; - Bi-layered SC/eNFs carrier showed the most proper physical architecture for proliferation and cell adhesion due to the highest value of cell viability measured in comparison with bi-layered SC/3D carrier; - High potential for the state of the art of technical approach with inkjet printing-electrospinning in fabrication of bioactive wound patches.	[121]
**SA/Poly lactic acid (PLA)/polyvinyl alcohol (PVA)**	**-**	Blend electrospinning	- In vitro studies demonstrated that PLA/PVA/SA nanofiber scaffold could offer proper anchor for the proliferation of rat fibroblasts (L929); - In vivo biological assessment was performed on skin defects rat models in which the formulated nonofibrous membranes improved wound healing with a reduction of the inflammatory reaction during incipient wound healing compared to commercially available gauzes.	[122]
**SA/PVA-Triton-Chitosan (CS)**	**Dex-Panthenol**	Blend electrospinning	- Drug release of dexpanthenol followed the Fickian diffusion mechanism with the model of Korsmeyer-Peppas. - Cell culture and MTT analysis revealed that dexpanthenol-loaded SA/PVA/Triton-CS eNFs were non-toxic towards fibroblast cells and improved the cellular attachment. - It was indicated that SA/PVA/Triton-CS eNFs can be utilized for various applications in tissue engineering.	[84]

**Table 3:** Recent research on cellulose derivatives-based electrospun nanofibers (C-eNFs) used as wound dressings.

Biopolymer/ Copolymer	Bioactive Agent	Type of Electrospinning	Main Results	References
**Cellulose acetate** **(CA)**	**Silver sulfadiazine (SSD)**	Blend electrospinning	- Morphology with uniform distribution of SSD in the CA/SSD eNFs mats; - Water contact angle assay and XRD spectra revealed proper water absorbency essential for scaffoldings; - CA/SSD eNFs indicated appreciable antibacterial effect against Gram-negative *Escherichia coli* and Gram-positive *Bacillus subtilis* strains; - Promising product for wound dressings applications.	[90]
**CA**	**Manuka honey (MH)**	Blend electrospinning	- MH incorporation into the CA-MH eNFs exhibited high efficiency to hinder bacterial growth on the wound area and good antioxidant capacity, dependent on the immersion time in the DPPH solution and MH content; - The high porosity (85–90%) and water vapor transmission rate values of 1950–2600 g/m^2^/day demonstrates great ability for wound breathability; - In vitro testing revealed elevated cyto-compatibility to NIH 3T3 cell line demonstarting to be efficient for facilitating wound healing.	[132]
**CA** **/Gelatine (Gel)**	**Berberine**	Blend electrospinning	- Berberine incorporation did not compromise the physical properties of nanofibrous dressing, but improved the biological activities. - Antibacterial assays demonstrated potent antibacterial activity; - The angiogenesis score of 19.8 ± 3.8 and collagen density of 88.8 ± 6.7% obtained in the streptozotocin-induced diabetic rats studies confirm a proper wound healing; - Potential wound dressing for diabetic foot ulcer (DFU) management and treatment.	[133]
**Ethyl cellulose (EC)** **/Polyvinyl** **pyrrolidone (PVP)**	**Ciprofloxacin (Cip)** **/silver nanoparticles (AgNPs)**	Side-by-side electrospinning process with acentric spinneret	- SEM analysis revealed a cylindrical, uniform morphology with a clear Janus structure and with AgNPs distribution in one side. - X-ray diffraction patterns outlined that Cip had an amorphous state due to fast drying and high compatibility with PVP; - In vitro assays showed a release of over 90% Cip within the first 30 min, concluding high antibacterial activity at the early phases of wound healing; - The formulated Janus eNFs indicated high bactericidal effect against the growth of both Gram-positive *S. aureus* and Gram-negative *E. coli*, ensuring promising candidate for efficient wound dressings.	[134]

**Table 4:** Recent discoveries on different gums-based electrospun nanofibers (G-eNFs) used as wound dressings.

Biopolymer/ Copolymer	Bioactive Agent	Type of Electrospinning	Main Findings	References
**Gum Tragacanth (GT)/PVA**	**-**	Blend electrospinning	- Differential Scanning Calorimetry (DSC) indicated that the exothermic peak at 194 °C for PVA has displaced at an inferior temperature in GT/PVA mix. - Good antibacterial activity of GT/PVA eNFs against Gram negative bacteria *(Pseudomonas aeruginosa)* and proper cell adhesion and proliferation on human fibroblast;	[144]
**Guar gum (GG)/PVA**	**Paramagnetic iron oxide Fe_3_O_4_ nanoparticles**	Blend electrospinning	- eNFs obtained from alkaline stock solutions had an increase homogeneity disposition of nanoparticles as a result of the beneficial interactions between the metallic ion and GG; - In vitro biocompatibility assays via L929 cells showed proper degrees of cytotoxicity and also cell adhesion and proliferation for both eNFs mats yielded from non-alkaline/alkaline stock solutions; - Feasible for pharmaceutical applications as biodegradable wound dressing.	[140]
**Iranian Gum Tragacanth (IGT)/PVA**	**Nano-clay powder (NC)**	Blend electrospinning	- The bio-ceramic nano-clay (NC) powder (1%, 3%) was added to improve the mechanical and chemical stability; - It was demonstrated that the elevated percentage of IGT confers superior mechanical, chemical stability, and degradation; - The scaffold based on NC-IGT/PVA eNFs 20/80 with 3% NC indicates an enhancement in their specific properties compared to pure IGT/PVA.	[96]
**Gum Tragacanth (GT)/poly(ε-caprolactone (PCL)**	**Curcumin (Cr)**	Blend electrospinning	- GT/PCL/Cr eNFs scaffolds have exhibit antibacterial property against methicillin resistant *Staphylococcus aureus*; - In vivo assessment was effectuated in healing full thickness wound on the dorsum of rats; the pathological test done after 15 day demonstrated that applying GT/PCL/Cr eNFs produces promptly wound closure with well-defined granulation tissue characterized by collagen accumulation, complete regenerated epithelial layer, fibroblast proliferation, together with sweat glands and hair follicles development; - Biomedical application of the formulated eNFs based on GT and loaded with curcumin for wound healing in diabetic rats.	[145]
**Gum Arabic (GA)-corn protein (Zein)/** **PCL**	**-**	Blend electrospinning	- SEM analysis indicated that GA/Zein/PCL eNfs scaffolds had a porous design with bimodal diameters dissemination, while, during its destruction it was revealed that the scaffold’s structure remains fibrous; - Properties like favorable porosity (approx. 80%), high hydrophilicity (about 80%) and tensile strength of 1.36–3MPa with an extension of 19.13–44.06% were desirable for skin tissue engineering. - In vitro test demonstrated prosperous L929 cells proliferation compared to control (tissue culture polystyrene) and antibacterial properties; - Potential application for skin tissue engineering to compensate deep skin damages.	[95]
**Gum Tragacanth (GT)** **/PCL-PVA**	**-**	Blend electrospinning	- Results indicated that the best ratio of GT:PCL:PVA is 2.2:2:0.8 for the formation of eNFS used for diabetic ulcers healing; - Mesenchymal stem cells on the eNFs based on GT indicated attachment and cells proliferation, while the histological analysis of substrates embodying stem cells from rats with diabetic ulcers demonstrated tissue repair/regeneration with collagen formation after 15 days; - Promising formulations for wound healing of diabetic ulcers (DU).	[141]
**Gum Arabic (GA)** **/PCL-PVA**	**Silver nanoparticles (AgNPs)**	Blend electrospinning	- It was proven antimicrobial action of eNFs mat against *Staphylococcus aureus*, *Pseudomonas aeruginosa*, *Escherichia coli*, and *Candida sp*; - Cytotoxicity assay of the nanofibrous mats indicated positive biocompatibility with the mouse embryonic fibroblast cells. - PCL-coated GA/PCL-PVA-AgNPs represent an efficient antimicrobial eNFs substitute for standard wound dressing.	[146]
**Gum Azivash** **(GAz)/PVA**	**Catechin (Cat)**	Blend electrospinning	- Higher catechin levels varying from 500–3000 mg L^−1^ exhibited a 5-fold increase in the loading capacity of GAz/PVA–Cat eNFs, - Cat entrapment in the inner structure of the eNFs improved the thermal resistance of the mats due to polymer interaction through hydrogen bonds and also increased the adhesion between molecular chains. - Good candidate for the design of scaffold for pharmaceutical applications, such as wound dressings.	[97]

**Table 5:** Overview over research on chitosan electrospun nanofibers (CS-eNFs) used as bio-degradable wound dressings.

Biopolymer/ Copolymer	Bioactive Agent	Type of Electrospinning	Significant Outcomes	References
**CS/PEO**	***Aloe vera***	Blend elctrospinning with Spirograph Based Mechanical System (SBMS) Collector	- Cell culture studies revealed increased cell proliferation and the lack of any cytotoxic action in the cell’s growth; - In vivo assays performed on mice model showed for *Aloe vera* incorporated electrospun mats a faster wound healing compared with the CS/PEO mats formulated with a standard static collector.	[150]
**CS/PEO**	**Bromelain** **(crude extract from pineapple)**	Blend electrospinning with rotating drum collector	- Low cytotoxicity using Alamar blue test for chitosan-2% *w/v* bromelain nanofibers than chitosan-4% *w/v* bromelain nanofibers; - The burn healing activity of CS-2% *w/v* bromelain eNFs analyzed for 21 days on the induced burn wounds in rats showed a reduction of burn wound area.	[151]
**CS/PVA**	**silk protein sericin (SS)/** **tetracycline** **(TC)**	Blend electrospinning	- The nanofibers loaded with silk sericin/tetracycline demonstrated outstanding bactericidal effect against both Gram-positive and Gram-negative bacteria; - L929 fibroblasts cultured on the eNFs with low sericin content displayed higher proliferation in comparison with those cultured on eNFs without sericin; - In vivo studies showed that CS/PVA/SS-TC eNFs increased re-epithelialization, wound healing, and collagen deposition in comparison with conventional gauze and with the eNFs without sericin.	[114]
**CS/SA**	**Gentamicin (Gn)**	Blend electrospinning	- In vitro cell culture assays showed that CS-SA wound dressings with 1–3% content of Gn enhanced L929 cell adherence and proliferation; - In vivo studies demonstrated that CS-SA eNFs loaded with 3% Gn improved skin regeneration in a Balb/C mice model by promoting the creation of a thicker dermis, and by intensifying the formation of novel hair follicles and blood vessels.	[99]
**CS-Gelatin (Gel)-Hyaluronic acid (HA)/PEO**	**-**	Dual spinneret electrospinning	- Higher cell proliferation for CS-Gel-HA/PEO eNFs (109%) in the first 24 h comparing with CS/PEO (90%) and CS-Gel/PEO (96%); - The in vivo wound healing findings performed on rat revealed more wound healing capacity of the CS-Gel-HA/PEO mat due to the formation of new tissue with a structure similar to that of normal skin.	[152]

**Table 6:** Recent studies on hyaluronic acid-based electrospun nanofibers (HA-eNFs) used for skin tissue regeneration.

Biopolymer/ Copolymer	Bioactive Agent	Type of Electro Spinning	Main Results	References
**HA/poly(lactic-*co*-glycolic acid) (PLGA)**	**Epigallo** **catechin-3-O-gallate (EGCG)**	Coaxial electro Spinning	- The amount of human dermal fibroblasts affixed on HA/PLGA-EGCG mats is significantly larger than that on HA/PLGA. - The wound healing potential of HA/PLGA-EGCG matrices is explored on streptozotocin-induced diabetic rats, where it was shown that the wound regions are appreciably decreased by the covering with HA/PLGA-EGCG demonstrating improved re-epithelialization/neo-vascularization and better collagen deposition, compared with HA/PLGA covering or no treatment.	[160]
**HA/PVA**	**2-Hydroxy** **propyl-beta-cyclodextrin/naproxen**	Blend electro Spinning	- After naproxen impregnation into the scaffolds in aqueous solution/under supercritical CO_2_ it was indicated a regular drug delivery profile through several days without altering the fibrous architecture.	[157]
**HA-Starch(S)/** **Polyurethane (PU)**	**-**	Coaxial electro Spinning	- In vitro assays using fibroblasts cells from mouse (L929) exhibited notable amelioration in cell attachment and cell differentiation; - The wound healing properties of the core-shell nanofibers were investigated using a wound excision rat model. It was observed a synergic activity of materials used in the development of HA-S/PU core-shell mats which justify the use as wound healing applications.	[88]
**HA-Polygalacturonic/Poly-vinyl alcohol (PVA)**	**Silver nanoparticles** **(AgNPs)**	Blend electrospinning with drum collector	- AgNPs demonstrated an anti-inflammatory and antioxidant activity that protects cells from the damaging effect of increased ROS and that also accelerates wound healing; - Significant inhibition zone of antimicrobial effect towards gram positive/gram negative bacterial strains; - In-vivo study on albino rat, had indicated, after 14 days of nanofibers administration, a maximum wound collagen deposition/epithelization.	[153]
**HA/PU**	**Ethanolic extract of propolis (EEP)**	Blend electros Pinning	- The best result in terms of biocompatibility for L929 fibroblast cells was found for HA-PU/1% EEP which displayed no cytotoxicity for the normal murine fibroblasts. - HA-PU/1% EEP scaffolds had produced substantial healing acceleration at the Wistar rats skin excisions model.	[103]

**Table 7:** Overview over research on Pullulan (PUL) nanofibers (PUL-NFs) used as potential material dressings.

Biopolymer/ Copolymer	Bioactive Agent	Type of Electrospinning	Main Results	References
**PUL** **/PVA**	**Rutin**	Blend elctrospinning	- SEM results indicated that adding rutin in a concentration higher than 8.54% (*w*/*w*) will form beaded eNFs and mechanical analysis showed that the tensile stress is directly proportional with the PVA ratio; - UV-resistant properties assay demonstrated that rutin incorporation was able to diminish the UVA and UVB transmittance to values lower than 5%, while the UPF value was above 40 and above 50 at a rutin concentration of 4.46% and 5.67%, respectively; - Feasible application as anti-ultraviolet dressing scaffolds.	[169]
**PUL** **/Sodium alginate (SA)**	**-**	Free-surface electrospinning	- Adding SA 0.8–1.6% (*w*/*w*) to a 10% (*w*/*w*) aqueous pullulan solution will lead to an expansion in polymer chain entanglement and to an enhanced hydrogen bonding connection between PUL and SA; - The addition of CaCl_2_ in trace amount (maxim 0.045%, *w*/*w*) was translated into ultrafine and smooth eNFs formation, characterized by a higher thermal stability than those formulated without adding CaCl_2_; - The water-based biopolymer systems formulation is useful for the development of nano-scale fibers used in various pharmaceutical applications.	[118]
**PUL** **/Sodium alginate (SA)**	**Human platelet lysate (PL)**	Blend electrospinning	- The PL entrapment in PUL/SA eNFs did not alter the eNFs morphology before crosslinking, while CaCl_2_ crosslinking determined less sharp eNFs; - The cytotoxicity assay revealed a random cell tendency in accordance with a fibroblast-to-myofibroblast conversion; - The formulated eNFs act as mats for tissue engineering with proper mechanical features and PL release, therefore, are suitable candidates for skin reparation dressings that could enhance wound healing.	[83]
**PUL/** **Chitosan (CS)**	**Tannic acid (TA)**	Forcespinning (FS)	- FS use centrifugal forces which permit a yield increase, ease of production, and a wider selection of materials to be spun as NFs; - The ternary NFs showed positive water absorption capacity with rapid uptake rate and with synergic antimicrobial effect towards Gram-negative bacteria (*Escherichia coli*); Also it has been revealed that by providing a 3D architecture which imitates skin’s ECM will allow fibroblast cell adherence and growth, so favoring prospective for intricate and deep wound healing.	[105]

**Table 8:** Studies conducted on Dextran (DXT) electrospun nanofibers (DXT-eNFs) processed as potential mats for wound management.

Biopolymer/ Copolymer	Bioactive Agent	Type of Electrospinning	Main Findings	References
**DXT** **/Polyurethane (PU)**	**Ciprofloxacin hydro** **chloride (Cip)**	Blend elctrospinning	- Cip addition decreased the size and narrowed down the partition of eNFs diameters, which was translated into a reduction in solution viscosity; - DXT inclusion into the PU enhanced the cell adherence and viability; - The DXT-PU-Cip eNFs showed good antibacterial potential towards both Gram-positive and Gram-negative bacteria; - A potential ideal antibacterial biomaterial for wound dressing applications.	[179]
**DXT-cellulose acetate (CA)** **/PCL**	**Tetracycline hydro-** **chloride (TC)**	Blend elctrospinning	- TC incorporation improved blood clotting, enhanced cell proliferation, cell attachment and the antimicrobial activity of DXT-CA-TC/PCL eNFs; - After fibroblast cells were seeded on the eNFs scaffolds, it was indicated a strongly increased cell attachment and proliferation; also, DXT-CA-TC/PCL eNFs exhibited high antibacterial activity, thanks to TC presence; - DXT-CA-TC/PCL eNFs present suitable properties for wound dressing development and skin engineering applications;	[181]
**DXT/PVA**	**Ciprofloxacin (Cip)**	Emulsion electrospinning	- DXT/PVA ratio blend was optimized and eNFs were stabilized by thermal treatment at 120 °C regarding disintegration in water; - SEM analysis correlated with DSC confirms the core-shell structure of the eNFs, while DSC indicated the DXT-PVA interaction; The in vitro release study showed a Cip sustain release, controlled by the DXT content which can hapen by diffusion within the delivery system; - DXT/PVA-Cip eNFs can be formulated by a green method and are auspicious eco-friendly drug delivery systems;	[106]
**DXT/PU**	**Curcumin (Cr)**	Blend elctrospinning	- DXT incorporation demonstrated increment in percentage sorption values, vapour transmission rate, biodegradability, and hydrophilicity; - DXT induces enhanced hemostasis potential and high degree of platelet adhesion essential for promoting wound healing; - 20 wt% DXT loaded eNFs (20DXT/PU) exhibited high attachment, cell proliferation and cytoviability against 3T3 fibroblasts; Cr loaded 20DXT/PU showed synergic antibacterial effect against Gram-positive bacteria and a pH-controlled drug release potency so it is promising wound dressing material.	[182]

## Abbreviations

AAD—adipic acid dihydrazide, Alg—alginate, AgNPs—silver nanoparticles, ATP—Adenosine triphosphate, BA—boric acid, C—cellulose, CA—cellulose acetate, Cat—catechin, CD44 — antigen encoded by the CD44 gene on Chromosome 11, CD 168 — hyaluronan acid-mediated motility receptor, Cip—ciprofloxacine hydrochloride, CMC—carboxymethyl cellulose, Cr—curcumin, CS—chitosan, Da—Dalton, DFU—diabetic foot ulcer, DMSO—dimethyl sulfoxyde, DPPH—2,2-diphenyl-1-picrylhydrazyl, DSC—Differential Scanning Calorimetry, DU—diabetic ulcer, DXT—dextran, EC—ethyl cellulose, ECM—extracellular matrix, EEP—Ethanolic extract of propolis, EGCG—epigallo catechin-3-O-gallate, EGF—epidermal growth factor, EGFR—epidermal growth factor receptor, eNFs—electrospun nanofibers, FDA—Food and Drug Administration, FGF—fibroblast growth factor, FT—IR-Fourier transform infrared spectroscopy, FS—Forcespinning, GA—gum Arabic, GAz—gum Azivash, Gel—gelatin, GFs—growth factors, GG—gum guar, GK—gum karaya, Gn—gentamicin, GT—gum tragacanth, HA—hyaluronic acid, HBSS—Hanks’ balanced salt solution, HIF-1—Hypoxia-inducible factor-1, HIF α—Hypoxia-inducible factor alfa, HBOT—hyperbaric oxygen treatment, IGF—insulin growth factor, IGT—Iranian Gum Tragacanth, IL-1—interleukin-1, MC—methylene chloride, MH—Manuka Honey, MTT—dye compound 3-(4,5-Dimethylthiazol-2-yl)-2,5-diphenyltetrazolium bromide, NADPH—nicotinamide adenine dinucleotide phosphate, NC—Nano-clay, NK cells—natural killer cells, PANI—polyaniline, PCL—poly (ε-caprolactone), PCT—pectinate, PDGF—platelet-derived growth factor, PEO—polyethylene oxide, PGF—platelet growth factor, PHDs—HIF-prolyl-4-hydroxylases, PL—Human platelet lysate, PLA—poly lactic acid, PLGA—poly (lactic-*co*-glycolic acid), PU—polyurethane, PUL—Pullulan, PVA—polyvinyl alcohol, PVP—polyvinylpyrrolidone, ROS—reactive oxygen species, S—starch, SA—sodium alginate, SBMS—Spirograph Based Mechanical System, SC—solvent cast, SEM—Scanning electron microscopy, SPG—Schizophyllan, SS—silk sericin, SSD—Silver sulfadiazine, TA—Tannic acid, TC—tetracycline hydrochloride, TFA—trifluoroacetic acid, TGA—Thermogravimetic analysis, TGF-α—transforming growth factors α, TGF-β—transforming growth factors β, TLR-2—Toll like receptor 2, TLR-4—Toll like receptor 4, TNF-α—tumor necrosis factor-α, UPF—ultraviolet protection factor, UVA—longest ultra violet wavelength, UVB—medium ultra violet wavelength, VEGF—vascular endhotelial factor, vWF—von Willebrand factor, XG—xanthan gum, XPS—X ray photoelectron spectroscopy, XRD—X-ray diffraction pattern, Zein—corn protein.
